# Conformer-Specific Dissociation Dynamics in Dimethyl Methylphosphonate Radical Cation

**DOI:** 10.3390/molecules27072269

**Published:** 2022-03-31

**Authors:** Vaibhav Singh, Hugo A. López Peña, Jacob M. Shusterman, Patricia Vindel-Zandbergen, Katharine Moore Tibbetts, Spiridoula Matsika

**Affiliations:** 1 Department of Chemistry, Temple University, Philadelphia, PA 19122, USA; vaibhav.singh@temple.edu; 2 Department of Chemistry, Virginia Commonwealth University, Richmond, VA 23284, USA; lopezpenaha@vcu.edu (H.A.L.P.); shustermaj@vcu.edu (J.M.S.); kmtibbetts@vcu.edu (K.M.T.); 3 Department of Physics, Rutgers University at Newark, Newark, NJ 07102, USA; pv.zandbergen@rutgers.edu

**Keywords:** strong field ionization, nonadiabatic dynamics, conical intersections, excited states, radical cation, conformers

## Abstract

The dynamics of the dimethyl methylphosphonate (DMMP) radical cation after production by strong field adiabatic ionization have been investigated. Pump-probe experiments using strong field 1300 nm pulses to adiabatically ionize DMMP and a 800 nm non-ionizing probe induce coherent oscillations of the parent ion yield with a period of about 45 fs. The yields of two fragments, PO_2_C_2_H_7_^+^ and PO_2_CH_4_^+^, oscillate approximately out of phase with the parent ion, but with a slight phase shift relative to each other. We use electronic structure theory and nonadiabatic surface hopping dynamics to understand the underlying dynamics. The results show that while the cation oscillates on the ground state along the P=O bond stretch coordinate, the probe excites population to higher electronic states that can lead to fragments PO_2_C_2_H_7_^+^ and PO_2_CH_4_^+^. The computational results combined with the experimental observations indicate that the two conformers of DMMP that are populated under experimental conditions exhibit different dynamics after being excited to the higher electronic states of the cation leading to different dissociation products. These results highlight the potential usefulness of these pump-probe measurements as a tool to study conformer-specific dynamics in molecules of biological interest.

## 1. Introduction

Many photoinduced biological [1,2,3] and chemical [4,5] processes require the understanding of nuclear and electronic dynamics which occur on nanosecond to attosecond time scales. Ultrafast pump-probe spectroscopy [6] has been an effective experimental technique to study the dynamics occurring on an ultrafast time scale. In particular, strong field ionization followed by dissociation has been a useful technique to probe ultrafast dynamics in radical cations [7,8,9,10,11,12]. The pump pulse here ionizes molecules to create radical cations, whose dynamics are then studied with the help of a weak probe pulse that can excite to higher electronic states of the cation to induce dissociation.

One of the challenges in strong field ionization is to prepare a well-defined coherent state in the radical cation without excessive molecular fragmentation [12,13]. Strong field ionization often creates a superposition of electronic states in the cation, leading to a high probability that multiple fragmentation pathways will be accessed to form different fragments in an uncontrolled manner. However, strong-field ionization at laser wavelengths in the near-infrared region (∼1200–1600 nm) can prepare a well-defined initial coherent state [9,13,14,15]. In the limit of adiabatic ionization, the strong field allows an electron to tunnel out through a Coulombic barrier, forming the parent cation on the ground electronic state, significantly reducing the number of fragmentations [14,16]. Adiabatic ionization is often described in terms of the Keldysh parameter (γ) [17], defined as the frequency of incident laser pulse divided over the electron tunneling frequency. When the laser frequency is sufficiently low, γ is less than 1 and adiabatic ionization occurs primarily through tunneling, as is evidenced experimentally by less fragmentation [9,13,14]. Higher laser frequency (shorter wavelength), on the other hand, results in γ greater than 1 where tunneling is diminished, leading to non-adiabatic ionization and high degree of fragmentation.

Preparation of radical cations in their ground state via adiabatic ionization has led into a number of interesting observations of ion yield oscillations arising from the initial coherent vibrational dynamics. Levis and coworkers observed that coherent ion yield oscillations in acetophenone cation lasted 100 fs longer and were six times amplified when acetophenone was ionized adiabatically at 1270 nm as compared to ionizing nonadiabatically at 800 nm [9,18]. The antiphase oscillations between the parent acetophenone and fragment benzoyl cations with 650 fs period were attributed to coherent motion along the phenyl-acetyl twisting coordinate. Similar coherent twisting motions upon adiabatic ionization have been observed in other aromatic molecules including azobenzene [19], nitrobenzene [20], and nitrotoluenes [11,21,22]. There are many such instances where adiabatic ionization improved the amplitudes and lifetimes of oscillations in the ion signals of parent cation and secondary fragments, which provided better insight into the dynamics [12].

In recent studies one of us applied adiabatic ionization on dimethyl methylphosphonate (DMMP, PO${}_{3}$(CH${}_{3}$)${}_{3}$) [13,23]. DMMP is a well-known simulant for organophosphorus chemical warfare agents, such as sarin and soman [24,25]. These nerve agents can lead to nerve paralysis and sometimes even death. Hence, having control schemes through which these molecules can be destructed might be helpful in detection of these agents. The experiment used near-infrared (1200 nm or 1500 nm) and 800 nm pump pulses to ionize DMMP adiabatically and non-adiabatically, respectively. Adiabatic ionization prepared a well-defined coherent vibrational state along the P=O stretching coordinate in the radical cation, resulting in high-amplitude ion yield oscillations with a period of 45 fs. The two major fragment ions, PO_2_C_2_H_7_^+^ and PO_2_CH_4_^+^, oscillate with the same period as the parent ion but with a phase shift of approximately π. These antiphase oscillations were attributed to electronic excitation of the parent ion by the 800 nm probe pulse, but no specific excitation pathways were identified.

Determining specific excitation pathways in DMMP${}^{+}$ is complicated by the fact that DMMP, like many molecules, is present at room temperature as multiple rapidly converting conformational isomers, or conformers. Conformer-specific ionization and reaction dynamics have been observed in many molecules through spectroscopic excitation over the last two decades [26,27,28,29,30,31,32,33]. Most of these studies first separate distinct conformers spectroscopically or electrostatically before probing their individual dynamics and reactions [26,27,28,29,30], although more recently advanced techniques including Coulomb explosion imaging [31] and ultrafast electron diffraction [32,33] have distinguished structures and reaction dynamics of multiple conformers without prior separation. However, the potential for conformer-specific coherent vibrational dynamics to result in distinct excitation and dissociation pathways remains unexplored.

In this work, we use computational studies in an effort to better understand and interpret the dynamics observed by pump-probe spectroscopy on DMMP^+^, including the different conformers that are expected to be present. Trajectory surface hopping used to obtain the dynamics of the prepared cationic states reproduces the oscillatory behavior seen experimentally. High level coupled cluster calculations are also used to calculate higher cationic states that can be accessed with the probe laser, which clarifies specific features of the pump-probe signals in different fragmentaion products. A comparison between a 800 nm and a 400 nm probe is also made providing further insight into the dynamics of DMMP^+^. The combination of the theoretical and experimental work offers a detailed description of the overall dynamics, and sheds light into how the excited state dynamics of different conformers can affect fragmentation.

## 2. Methods

### 2.1. Electronic Structure Calculations

All the geometries were optimized using Density Functional Theory (DFT) [34,35] with the B3LYP [36,37,38,39] functional and the 6-311+G(d) [40,41,42,43,44] basis set available in Gaussian 09 suite of packages [45]. Several conformers were optimized using initial structures reported previously, and were reoptimized in the current work for consistency [46]. Only two of these conformers are present at the experimental conditions, as will be discussed later. One of these conformers has C${}_{s}$ symmetry while the other one has no symmetry and will be denoted as C${}_{1}$. The neutral and the cationic geometries refer to the ground state minima of the neutral DMMP (S_0_ minimum) and the radical cation DMMP^+^ (D_0_ minimum) respectively. The single ionization potentials (IPs) of neutral DMMP were calculated at these optimized geometries at the EOM-IP-CCSD/6-311+G(d) level of theory. The IP calculations were also repeated with larger Dunning’s correlation consistent basis set cc-pVTZ [47,48] to check the accuracy of the 6-311+G(d) results. Results are compared in Supplementary Material (SM) (Table S2), and confirm the accuracy of the 6-311+G(d) basis set.

In order to estimate the potential energy surfaces connecting the optimized neutral and cationic geometries, linear interpolations in all internal coordinates (LIIC) were done with 5 geometries connecting the initial and final geometries. Excited state energies and oscillator strengths of the transitions from the ground state (D_0_) to the four excited states of DMMP cation were then calculated along the LIIC paths using EOM-EE-CCSD/6-311+G(d), CASSCF and EOM-IP-CCSD/6-311+G(d). In order to calculate the oscillator strengths from the first excited state to the higher states, the multi-reference method CASSCF [49] with an active space of 13 electrons in 9 orbitals and averaged over 7 states (7SA-CAS(13,9)) was used. For the active space of CASSCF, the P=O π orbitals and the lone pairs of oxygens were included. The orbitals are shown in SM (Figures S6 and S7). The active space was chosen by performing initial benchmarking studies to determine the best active space able to describe the first 5 IP states (See SM, Table S3). These CASSCF calculations were done with 6-311+G(d) basis set.

Conical intersections (CoIns) between cationic states, D${}_{1}$/D${}_{2}$ and D${}_{2}$/D${}_{3}$, were calculated in order to explore pathways that facilitate radiationless decay after the probe excites population to higher states. Minimum points on the seam of CoIns were optimized using 5SA-CASSCF(13,9) with the 6-311G(d) basis set. We had to reduce the average of states from 7 to 5 and remove the diffuse functions because it was difficult to converge the calculations. The COLUMBUS software was used for the optimizations [50,51,52]. Because of the flexibility of the molecule it was not always possible to converge at the minimum of the seam. In that case we considered geometries on the seam (degenerate energies) even if the minimum was not reached. The energies at these geometries were recalculated using EOM-IP-CCSD/6-311+G(d).

Formation of two major fragments observed experimentally with *m*/*z* ratio 94 and 79 atomic mass require a hydrogen transfer step before fragmentation can occur. For this reason we calculated a pathway for the hydrogen transfer process. The optimized geometry of the tautomer produced from hydrogen transfer (denoted as HT) and the transition state connecting it to minimum of the ground state of DMMP${}^{+}$ (denoted as TS${}_{HT}$) were optimized using B3LYP/6-311+G(d). The pathway connecting the conical intersections to TS${}_{HT}$ was calculated using a LIIC and calculating the energies of the five ionic states at the EOM-IP-CCSD/6-311+G(d) level of theory. These LIIC provide a path after the probe excitation to reach the intermediate HT which is needed for fragmentation to occur.

All CCSD calculations were done using Q-Chem suite of packages [53], while CASSCF calculations were performed using MOLPRO suite of packages [54]. The conical intersection searches were performed using COLUMBUS [50,51,52].

### 2.2. Dynamics

Generally, the strong field adiabatic ionization populates the ground state of the radical cation but since the first two IPs at S_0_ geometry are very close, there is a high probability of the first excited state of DMMP^+^ (D_1_) to get populated along with D_0_ by the pump laser. Hence, the dynamics on both of the states were studied using Trajectory Surface Hopping (TSH) [55,56]. We initially tested the behavior of the two dominant conformers by performing dynamics for both of them using one trajectory with zero initial momentum in each case. The results are shown in SM (Figure S20), and they demonstrate that the dynamics are very similar between the two conformers. Specifically, the main P=O vibration governing the oscillations on D${}_{0}$ is identical for the two conformers. This is expected given the fact that the vibrational frequency for that mode is very similar for the two conformers (771 and 726 cm${}^{-1}$ for C${}_{s}$ and C${}_{1}$ conformers, respectively). For this reason the subsequent dynamics, where we use many trajectories for a statistically significant picture, were performed only on one of the conformers, the C${}_{1}$.

To mimic the wave packet at t = 0 fs (when DMMP is ionized), 100 initial geometries (initial conditions) and their kinetic energies were generated around the S_0_ minimum of the C${}_{1}$ DMMP using a harmonic oscillator Wigner distribution, as implemented in Newton-X [57]. Wigner distribution requires the S_0_ geometry and its normal modes to create these initial conditions; normal modes were calculated at the DFT level with the B3LYP functional and 6-311+G(d) basis set. The initial conditions were then propagated along the D_0_ and D_1_ potential energy surfaces (PESs) semiclassically: nuclei motions were treated classically with Newton’s equations of motion whereas electronic energies, gradients of PES along which these geometries evolved, and non-adiabatic couplings in between the surfaces were treated quantum mechanically. The velocity-verlet algorithm was used to deal with nuclear motion with a time step of 0.5 fs. Electronic energies, gradients and non-adiabatic couplings were calculated on-the-fly using a 2 states averaged CASSCF, (2SA-CAS(13,9))/6-311G(d) using COLUMBUS [50,51,52]. The same set of active space orbitals were used for CASSCF as prescribed in Section 2.1 above. The Fewest Switches Surface Hopping (FSSH) algorithm [58], as implemented in Newton-X 2.2 [59], was used to consider the hopping between electronic surfaces. The dynamics were run for 200 fs. In order to conserve the total energy after a hop, the momentum vector of the nuclei was re-scaled along the derivative coupling vector. To deal with the decoherence of the wave functions after a hop, the Persico and Grannuci approach [60] was used, with the suggested decoherence factor of 0.1 Hartree [61]. Also, the trajectories were killed when the total energy deviated by 0.5 eV or more when compared with the total energy at the previous time step or at the time t = 0 fs.

### 2.3. Experimental Methods

The pump-probe experimental setups have been described in detail in our previous work [22,62]. Briefly, DMMP (Sigma-Aldrich) introduced into the vacuum chamber of a time-of-flight mass spectrometer was ionized with a 1300 nm, 20 fs, $8\times 10^{13}$ W cm${}^{-2}$ pump pulse. Separate measurements with two different probe pulses were performed. The first measurement with 800 nm, 35 fs, $8\times 10^{12}$ W cm${}^{-2}$ probe pulses and a time step of 3 fs used the setup described in Ref. [62]. The second measurement with 400 nm, 70 fs, $4\times 10^{12}$ W cm${}^{-2}$ pulses and a time step of 10 fs used the setup described in Ref. [22].

## 3. Results

### 3.1. Experimental Motivation

**Figure 1:**
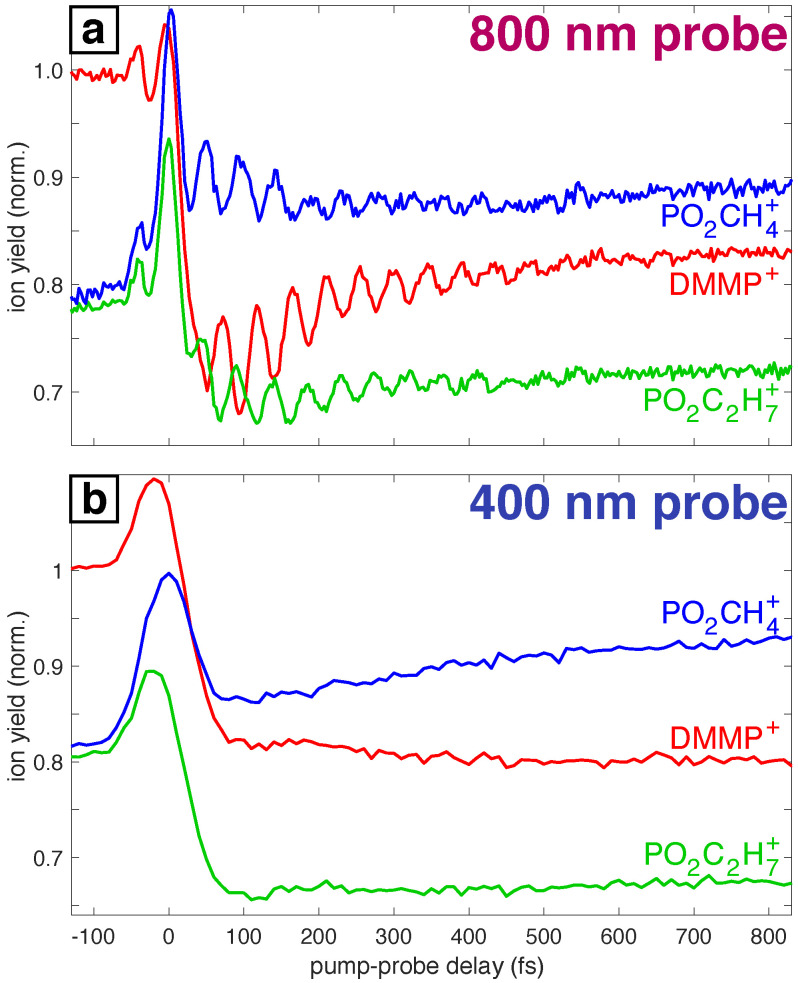
Transient ion signals of DMMP${}^{+}$ (red), PO_2_C_2_H_7_^+^ (green), and PO_2_CH_4_^+^ (blue) taken with (**a**) 800 nm and (**b**) 400 nm probe pulses.

Pump-probe measurements on DMMP with strong-field adiabatic ionization at 1300 nm were taken using probe pulses at both 800 nm and 400 nm. Here, we focus on the time-dependent yields of the parent DMMP${}^{+}$ ion and the two major fragments PO_2_C_2_H_7_^+^ (m/z 94) and PO_2_CH_4_^+^ (m/z 79). Previous mass spectrometry studies have established that PO_2_C_2_H_7_^+^ is produced directly from DMMP${}^{+}$ and that PO_2_CH_4_^+^ is formed from secondary dissociation of PO_2_C_2_H_7_^+^ [63]. The pump-probe results for DMMP using 800 nm probe are shown in Figure 1a. The approximately antiphase ion yield oscillations between the parent DMMP${}^{+}$ and fragments PO_2_C_2_H_7_^+^ and PO_2_CH_4_^+^ have been attributed to coherent excitation of the P=O stretching mode [13]. Moreover, the ion signal of DMMP${}^{+}$ substantially depletes to ∼70% of its negative delay value at around 100 fs, after which the signal increases to ∼84% of its original value within 800 fs. Both the oscillations and slower dynamics of the DMMP${}^{+}$ signal were observed in our earlier work [13,23]. The second measurement with 400 nm, 70 fs, $4\times 10^{12}$ W cm${}^{-2}$ pulses and a time step of 10 fs used the setup described in Ref. [22]. The transient ion signals from this measurement shown in Figure 1b do not exhibit the oscillations seen in Figure 1a because the long duration of the 400 nm probe pulse (70 fs) arising from frequency-doubling cannot resolve the coherent oscillations. Moreover, the DMMP${}^{+}$ signal rapidly depletes to ∼80% of its original value by 100 fs and remains constant thereafter, in contrast to the increase of DMMP${}^{+}$ signal over 800 fs seen with the 800 nm probe.

**Figure 2:**
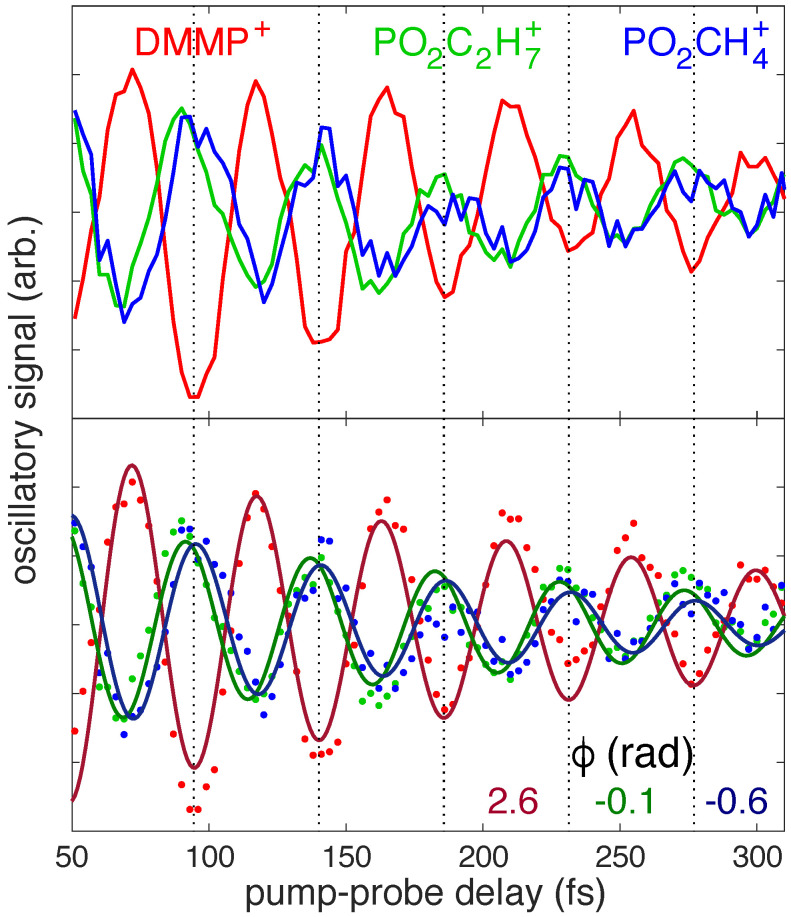
Oscillatory ion signals of DMMP${}^{+}$ (red), PO_2_C_2_H_7_^+^ (green), and PO_2_CH_4_^+^ (blue), shown as raw signals (**top**) and fit to exponentially decaying cosine functions (**bottom**). The extracted phases for each signal are shown; all signals had an oscillation period of 45.5 fs.

To isolate the oscillatory dynamics seen in Figure 1a, the dynamics at >50 fs delays were fit to a series of decaying exponential functions as described in ref. [23]. In DMMP${}^{+}$, the two decay times of (19 ± 9) fs and (177 ± 13) fs extracted from the incoherent dynamics (i.e., not associated with oscillations) may be associated with electronic relaxation. However, we cannot assign these time scales to any specific pathway because only the D${}_{0}$ and D${}_{1}$ dynamics were studied theoretically in this work. Subtracting off these incoherent dynamics isolates the oscillatory dynamics shown in Figure 2. In our previous work using 5 fs time steps, we reported that oscillations in the fragment ion yields were out of phase with the DMMP${}^{+}$ oscillations [13,23]. The present measurements taken with finer 3 fs time steps clearly show that the delays corresponding to the minima of the DMMP${}^{+}$ oscillations, highlighted with the dotted lines in Figure 2, do not *exactly* match the delays associated with maximum PO_2_C_2_H_7_^+^ or PO_2_CH_4_^+^ yields. Specifically, the PO_2_C_2_H_7_^+^ maximum (green) appears slightly ahead of the DMMP${}^{+}$ minimum (red) and the PO_2_CH_4_^+^ maximum (blue) appears slightly behind (Figure 2, top). To quantify these slight phase shifts, the oscillatory signals were fit to exponentially decaying cosine functions (Figure 2, bottom). The extracted oscillation phases of the PO_2_C_2_H_7_^+^ and PO_2_CH_4_^+^ yields differ by 0.5 radians, or about 8%. Their phases relative to the DMMP${}^{+}$ yield are 2.7 radians for PO_2_C_2_H_7_^+^ and 3.2 radians for PO_2_CH_4_^+^, demonstrating that neither fragment oscillates exactly out of phase (π radians) with DMMP${}^{+}$. This result could suggest distinct DMMP${}^{+}$ geometries preferentially dissociate into PO_2_C_2_H_7_^+^ or PO_2_CH_4_^+^ upon excitation at 800 nm.

### 3.2. Ionization Potentials

**Figure 3:**
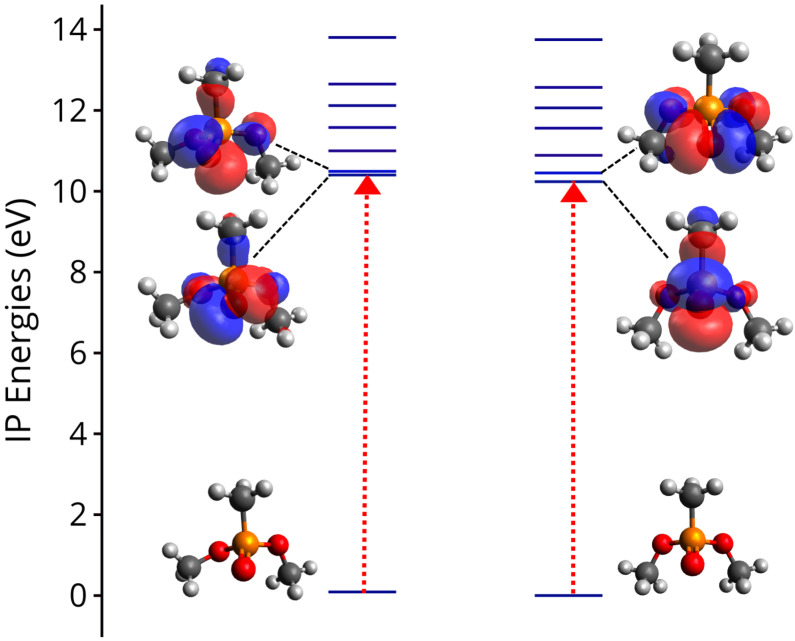
Vertical ionization potentials (IPs) of DMMP calculated at the EOM-IP-CCSD/6-311+G(d) level of theory for the C${}_{1}$ conformer (S_0,C${}_{1}$_ geometry, **left**) and C${}_{s}$ conformer (S_0,C${}_{s}$_ geometry, **right**). All energies are in eV and are plotted with respect to S_0,C${}_{s}$_ energy. The Dyson orbitals characterizing the hole of the first two cationic states are also shown, while the Dyson orbitals for the other states are shown in SM (Figures S4 and S5).

In order to understand the underlying dynamics of the DMMP cation we performed a series of computations, starting from the ionization energies to produce the cation in its various electronic states. Several conformers of DMMP and its cation have been reported before [46,64]. The conformers of neutral DMMP and their associated relative energies are shown in SM (Figure S3). Based on their energies, only two of them are expected to be present at the experimental conditions, while the others are expected to be approximately 2% present. The major distinction between these two nearly isoenergetic conformers can be made based on the two ‘O=P-O-C’ dihedral angles which are equal for one case leading to C${}_{s}$ symmetry, but vary by approximately 25${}^{\circ }$ for the other. The neutral equilibrium strcutures are denoted S_0,C${}_{s}$_ and S_0,C${}_{1}$_, respectively throughout the text (See Figure 3).

Figure 3 shows ionization potentials (IPs) to several cationic states calculated for both conformers. The first two states, D${}_{0}$ and D${}_{1}$, are almost degenerate, especially for the C${}_{1}$ conformer. This is because of the character of the two states, which is shown in the figure by their Dyson orbitals. The two states originate by ionization of an electron in orbitals located along the P=O bond, and there are two such orbitals in perpendicular planes. This explains why these two states are very close in energy and they behave very similarly, in consequence this will play an important role during the dynamics to be discussed next. Above D${}_{0}$ and D${}_{1}$ there are four additional states which are almost equally separated by about 1 eV from each other. The variation of these states as the molecule relaxes to the D${}_{0}$ minimum will play an important role in the observed pump probe behavior. The Dyson orbitals describing these states are shown in SM (Figures S4 and S5).

### 3.3. Dynamics on D${}_{0}$ and D${}_{1}$

**Figure 4:**
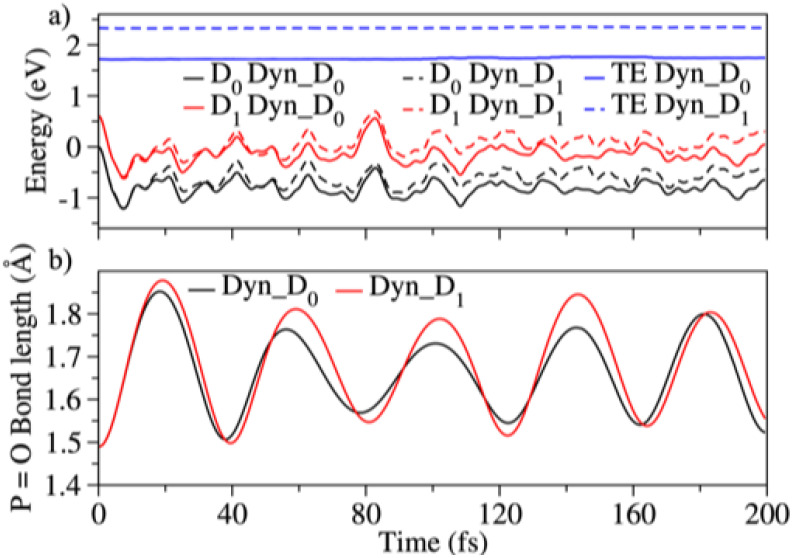
(**a**) Mean potential energies of D_0_, D_1_ and the total energies (TE) plotted vs time for the dynamics of the populations starting on the electronic states D_0_ (Dyn_D_0_) and D_1_ (Dyn_D_1_). The energies were calculated on-the-fly using 2SA-CAS(13,9)/6-311G(d) level of theory. (**b**) Mean P=O bond lengths plotted vs time for the dynamics starting on D_0_ or D_1_.

Generally, strong field adiabatic ionization creates a substantial population of a radical cation on its ground state [9]. However, for DMMP since the first two cationic states (D_0_ and D_1_) are almost degenerate, the probability of ionizing to D_1_ along with the ground state of DMMP^+^, D_0_, is high. Hence, to understand the dynamics after ionization, both the D_0_ and D_1_ states have to be considered. As mentioned in Section 2, we only show results for 200 trajectories run using the C${}_{1}$ conformer here. Comparisons for one trajectory between the C${}_{s}$ and C${}_{1}$ conformer are shown in SM (Figure S20), demonstrating very similar behavior. Figure 4 shows the main results from two different sets of dynamics, one where all the population is on D${}_{1}$ (Dyn_D_1_) and one with all the population on D${}_{0}$ (Dyn_D_0_). The mean energies of D_0_ and D_1_ for all the trajectories initially populated on D_0_ (Dyn_D_0_) or D_1_ (Dyn_D_1_) are shown plotted versus time in Figure 4a. In Figure 4b the mean P=O bond length versus time for both sets of trajectories is shown. We plot the P=O bond length vs time because this is a main distortion going from the neutral geometry to the relaxed D${}_{0}$ minimum, so this internal coordinate is a main evolving coordinate during the dynamics. This is also apparent by the nature of the orbital describing the unpaired electron which involves the P=O π bond for both D_0_ and D_1_ (Figure 3).

We first observe that the energies and P=O bond lengths for the two states D_0_ and D_1_ are parallel to each other for both sets of trajectories. This indicates that the potential energy surfaces of the states are parallel to each other and behave exactly the same way, so the dynamics observed are not affected by the fact that both states are populated. When the population starts in D${}_{1}$ there is fast decay to D${}_{0}$ (as shown in SM, Figure S17), but the nonadiabatic transitions do not affect the dynamics, as is clear from the oscillation of the P=O bond.

The most important observation is that the P=O bond length has an oscillatory behavior with time with a period of approximately 40 fs. Once the DMMP^+^ cation is created populations on either D_0_ or D_1_ oscillate along the P=O stretch with the time period of 40 fs. This period is very similar to the experimentally observed period in the fragments, and as will be discussed below is responsible for the experimental oscillations.

The fact that there are two conformers may complicate the dynamics, especially if conversion between them is observed. The barrier to convert between them is only 0.14 eV (see SM, Figure S12). In the dynamics however, we did not observe any meaningful conversion. Only two out of the total 200 trajectories converted from C${}_{1}$ to the C${}_{s}$ conformer during the dynamics. Figure S19 in the SM demonstrates this by showing the average of the two dihedral angles C-O-P-O, which never become equal (as they should be in the C${}_{s}$ conformer). This suggests that the C${}_{1}$ conformer remains asymmetric during the dynamics and similar behavior should be expected from the dynamics of the C${}_{s}$ conformer.

### 3.4. Effect of the Probe: Accessing Higher Electronic States

**Figure 5:**
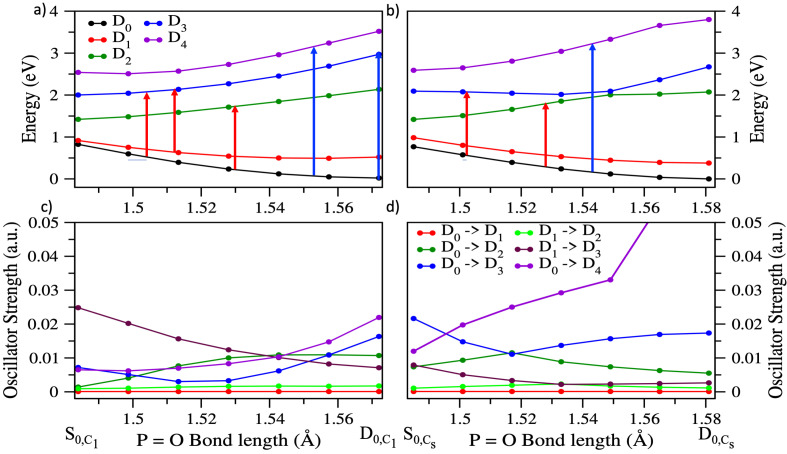
Energies along LIIC connecting vertical ionization to the minimum of the cation for (**a**) the asymmetric conformer, connecting the neutral minimum geometry, (S_0,C${}_{1}$_) to the cation minimum geometry (D_0,C${}_{1}$_); (**b**) the symmetric conformer, connecting the neutral minimum geometry (S_0,C${}_{s}$_) to the cation minimum geometry (D_0,C${}_{s}$_). The transitions due to probe light of 400 nm (3.1 eV) and 800 nm (1.6 eV) are represented with blue and red arrows respectively. (**c**,**d**) Oscillator strengths along the paths shown in (**a**,**b**), respectively. The vertical energies were calculated at the EOM-IP-CCSD/6-311+G(d) level of theory. The oscillator strengths were calculated at the 7SA-CASSCF(13,9)/6-311+G(d) level of theory.

Using the results from the dynamics we can explain how the probe is responsible for the oscillatory behavior in the ion signals of the parent cation and the secondary fragments. Once DMMP^+^ is formed due to the pump pulse, the wavepacket oscillates on D_0_ and D_1_ with the time period of about 40 fs. The probe pulse then excites the population either from D_0_ or D_1_ to the higher electronic excited states depending on certain conditions: the energy gap between D${}_{0}$ or D${}_{1}$ and higher states has to be resonant with the probe energy, and the oscillator strength between the resonant states has to be non-negligible. To get a better picture of which excited states are populated by the probe we calculated the PES of several electronic states along the oscillatory coordinate. In Figure 5 the energies of several states are plotted along the geometries generated with linear interpolation connecting the neutral geometry (corresponding to vertical ionization) to the minimum of the cation. Since, there was no evidence of conversion between the C${}_{1}$ and C${}_{s}$ conformers in the dynamics, we use LIICs connecting the C${}_{1}$ neutral minimum geometry (S_0,C${}_{1}$_) to C${}_{1}$ D_0_ minimum geometry of the cation (D_0,C${}_{1}$_) and the C${}_{s}$ neutral minimum geometry (S_0,C${}_{s}$_) to the C${}_{s}$ D_0_ minimum geometry (D_0,C${}_{s}$_) separately. The results at the EOM-IP-CCSD level are shown in Figure 5a,b, for C${}_{1}$ and C${}_{s}$, respectively, while similar plots at the CASSCF and EOM-EE-CCSD level are shown in SM (Figures S8 and S9). The oscillator strengths along the LIICs are plotted in Figure 5c,d, for the C${}_{1}$ and C${}_{s}$ conformers, respectively. Oscillator strengths are taken from the CASSCF calculations since EOM-IP-CCSD cannot calculate them between pairs of cationic states.

The electronic excited states that are accessible due to probe light are shown with red and blue single headed arrows representing the 800 nm (1.55 eV) and 400 nm (3.10 eV) probes, respectively. First, we discuss the effects of 800 nm probe light which leads to oscillations in the ion signals of the radical cations. For the 800 nm probe, the accessible states based on both the energy gaps and oscillator strengths are D_2_ or D_3_ for both conformers, as can be seen in Figure 5a,b. These transitions can cause depletion in DMMP^+^ population through dissociation into the observed fragments. Hence, the minima in the oscillation of DMMP^+^ ion signal occur at roughly the same time as the maxima in the ion signals of PO_2_C_2_H_7_^+^ and PO_2_CH_4_^+^ after every 40 fs. Experimentally, the time period of these oscillations is 45 fs (Figure 1) accounting for the relative error of our calculations to be 11%. This error is most likely due to the electronic structure. The CASSCF method that we used does not include dynamical correlation, so the structures and vibrational frequencies predicted are not very accurate. Errors in vibrational frequencies will directly affect the oscillation time.

With the 400 nm probe the higher energy states D${}_{3}$ and D${}_{4}$ can be accessed from D_0_ as shown by the blue arrows in Figure 5a,b. Also, the oscillator strengths for these transitions are comparatively higher than for the 800 nm transitions. Since multiple electronic states are accessible and the probe light of 400 nm provides extra energy, different pathways could be accessed. The excited states can dissociate into many other secondary fragments observed in higher yields in the mass spectrum at +800 fs delay with the 400 nm probe (SM Figure S1). Moreover, the resonance of the 400 nm probe with allowed transitions to D${}_{3}$ and D${}_{4}$ at geometries close to D${}_{0,C}{}_{1}$ and D${}_{0,C}{}_{s}$ is consistent with the continued depletion of the DMMP${}^{+}$ signal and increase in PO_2_CH_4_^+^ at a time delay of +800 fs for the 400 nm probe seen in Figure 1. In contrast, the lack of resonant transitions with the 800 nm probe near the D${}_{0,C}{}_{1}$ and D${}_{0,C}{}_{s}$ geometries explains the observed increase in intact DMMP${}^{+}$ signal with the 800 nm probe as the delay increases from 100 to 800 fs.

### 3.5. Differences between PO_2_CH_4_^+^ and PO_2_C_2_H_7_^+^: Conformational Effects

Based on the previous discussion we expect that the fragments PO_2_CH_4_^+^ and PO_2_C_2_H_7_^+^ are generated after probe excitations to D${}_{2}$ and D${}_{3}$ when the population of the parent ion is depleted. The oscillatory behaviors of these fragments in Figure 2 however are not completely in phase with one another. There is a small shift between them with PO_2_C_2_H_7_^+^ appearing slightly earlier than PO_2_CH_4_^+^. According to our theoretical results there are two sources that can lead to this difference. The first hypothesis is that the different fragments are associated with excitation to separate excited states. According to Figure 5, the D${}_{3}$ state can be accessed at shorter P=O bond lengths, which would imply that excitation to D${}_{3}$ leads to PO_2_C_2_H_7_^+^ and excitation to D${}_{2}$ leads to PO_2_CH_4_^+^. The second hypothesis is that the different conformers, C${}_{1}$ and C${}_{s}$, are responsible for the two different fragments.

**Figure 6:**
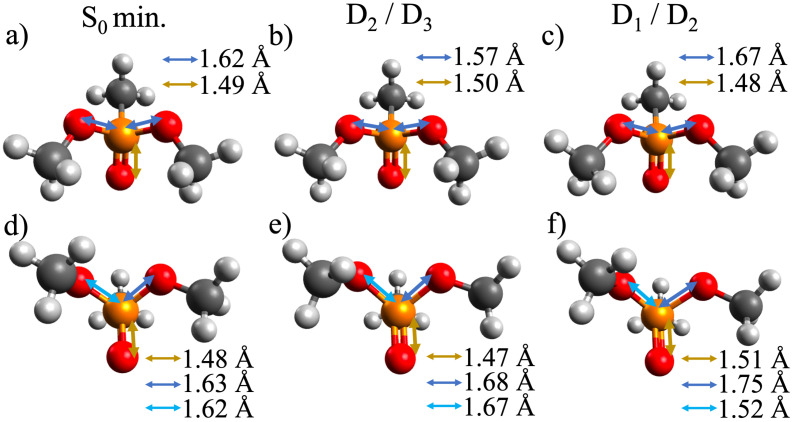
Structures of (**a**,**d**) S${}_{0,C}{}_{s}$ and S${}_{0,C}{}_{1}$ geometries, and the conical intersections between (**b**,**e**) D${}_{2}$/D${}_{3}$ and (**c**,**f**) D${}_{1}$/D${}_{2}$ states for the (**b**,**c**) C${}_{s}$ symmetric and (**e**,**f**) C${}_{1}$ asymmetric conformers. CoIns structures are optimized at the CASSCF level. The three P-O bond lengths are shown in Å.

In order to test these hypotheses we need to examine the dynamics leading to dissociation. The dynamics should either be different between the two excited states or between the two conformers. The most likely pathway for fragmentation is that internal conversion to the ground state converts the electronic energy to extra vibrational energy which can be used to break bonds. Internal conversion should be very fast because of the close proximity of the states. Hence, after the probe excitation, the population on D${}_{3}$ or D${}_{2}$ will decay very fast to D${}_{0}$. Fast decay among cationic states has been calculated before for other systems [10], and is expected to be common in radical cations due to the high density of states. The radiationless transitions between pairs of states will lead to certain modes becoming vibrationally excited. In order to have a better idea of how the dynamics will proceed we calculated conical intersections between D${}_{2}$/D${}_{3}$ and between D${}_{1}$/D${}_{2}$. The structures of these CoIns are shown in Figure 6. The main deformations occur along the two P-O bonds connected to the methyl groups. The third P=O bond that is responsible for the dynamics on D${}_{0}$ and D${}_{1}$ remains mostly unchanged at the initial value from vertical ionization of about 1.5 Å. Hence, any dynamics initiated by excitation to D${}_{3}$ and D${}_{2}$ will lead to vibrational excitation on the two P-O bonds. The branching vectors of the CoIns (shown in SM, Figure S13) show similarly that there is a lot of vibrational motion along the P-O bonds for both D${}_{2}$/D${}_{3}$ and between D${}_{1}$/D${}_{2}$ CoIns. These observations do not lead to any obvious differences between motion initiated on D${}_{3}$ vs D${}_{2}$. On the other hand, there are some obvious differences between the C${}_{s}$ and C${}_{1}$ geometries of the CoIns. In the C${}_{s}$ conformer the D${}_{2}$/D${}_{3}$ CoIn leads to a small contraction of the P-O symmetric bonds from their initial value of 1.62 Å to 1.57 Å. The D${}_{1}$/D${}_{2}$ CoIn though increases these bonds to 1.67 Å creating a vibrationally excited motion along these bonds. The C${}_{1}$ conformer behaves the opposite way. The D${}_{2}$/D${}_{3}$ CoIn increases the P-O bonds to 1.67 Å while the D${}_{1}$/D${}_{2}$ CoIn leads to a very asymmetric structure with one bond contracted and the other extended significantly. So, in this structure it is more likely that vibrational excitation is mostly on one P-O bond. It is natural to expect then that this asymmetric deformation can easier lead to a fragmentation where only one P-O bond is broken (as in PO_2_C_2_H_7_^+^) while the C${}_{s}$ conformer with its symmetric expansion of the P-O bonds can lead to excess vibrational energy on both P-O bonds which can eventually lead to the sequential fragmentation producing PO_2_CH_4_^+^.

Although these calculations provide a clear correlation between the two conformers and the two observed fragments, it is harder to explain the appearance of fragment PO_2_C_2_H_7_^+^ at slightly earlier times. This will depend on when exactly the gap between D${}_{0}$ and D${}_{3}$ matches the photon energy in the two conformers. The energy difference is very sensitive to the level of theory we are using, so we cannot be confident that we can resolve very small changes. On the other hand, the different behavior of the D${}_{3}$ state between the two conformers that can lead to different fragments is observed at all levels of theory we used. As seen in Figure 5 the slope of D${}_{3}$ is very different in the two conformers, with D${}_{3}$ increasing in energy along the oscillations in the asymmetric conformer and decreasing in the symmetric conformer. This is also observed using CASSCF and EOM-EE-CCSD, as seen in SM (Figures S8 and S9). Overall, the calculations support with reasonable confidence the assignment that excitation of the C${}_{1}$ conformer will lead to PO_2_C_2_H_7_^+^ while excitation of C${}_{s}$ leads to fragment PO_2_CH_4_^+^.

### 3.6. Hydrogen Transfer

Dissociation on the ground state to form the observed fragments PO_2_C_2_H_7_^+^ and PO_2_CH_4_^+^ requires an initial hydrogen transfer step. In the HT isomer, one of the hydrogens from the oxymethyl group migrates to the oxygen attached to phosphorus. We have located a transition state on D${}_{0}$ which can lead to this HT isomer (denoted TS${}_{HT}$). This transition state is the same for both conformers and we have connected it to the HT conformer (SM Figure S14).

**Figure 7:**
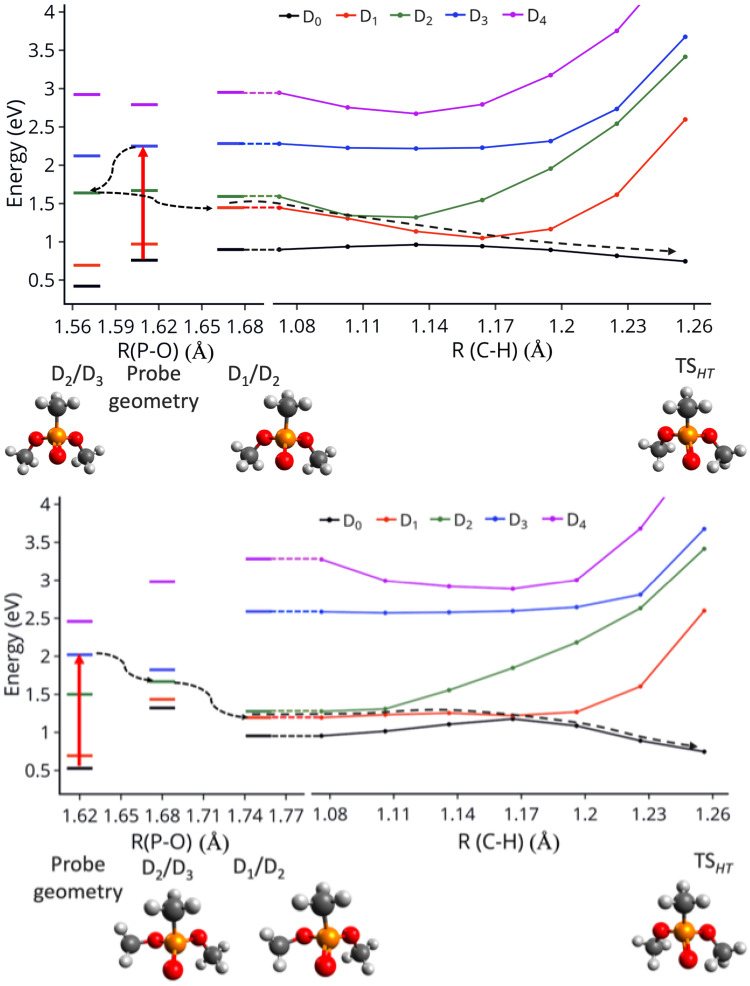
**Top**: Energies at the geometry of probe pulse excitation to D${}_{3}$; at the CoIn D${}_{2}$/D${}_{3}$ geometry and at the CoIn D${}_{1}$/D${}_{2}$ geometry. **Bottom**: Energies along LIICs connecting the D${}_{1}$/D${}_{2}$ CoIn to the transition state (TS${}_{HT}$). Top plots show the energies for the C${}_{s}$ conformer and bottom plots for the C${}_{1}$. All the calculations were done at EOM-IP-CCSD/6-311+G(d) level of theory.

Figure 7 shows how TS${}_{HT}$ can be easily accessed after the probe excitation for both conformers. On the top side, it is shown that after the probe excites the cation to D${}_{3}$ or D${}_{2}$, two CoIns D${}_{2}$/D${}_{3}$ and D${}_{1}$/D${}_{2}$ can be reached with the P-O bonds being the primary changing coordinates. On the bottom side of the figure, a LIIC connects the D${}_{1}$/D${}_{2}$ CoIn to TS${}_{HT}$. It is obvious from these figures that TS${}_{HT}$ is accessed barrierlessly after internal conversion to D${}_{1}$. A CoIn between D${}_{1}$ and D${}_{0}$ occurs along this path as well, as can be seen in Figure 7. The pathway is barrierless for both conformers, although it appears more downhill for the C${}_{s}$ conformer. The steeper slope for the C${}_{s}$ conformer can be associated with more excess vibrational energy in that conformer that can further be used for sequential fragmentation to PO_2_CH_4_^+^, which requires approximately 1 eV more energy than fragmentation to PO_2_C_2_H_7_^+^ [64].

## 4. Discussion

Conformationally selective dynamics are difficult to observe since it is often challenging to distinguish between conformers due to the small rotational barriers separating them. Nevertheless, the ubiquity and importance of conformers in chemistry and biology has inspired many experimental studies using a variety of techniques to observe conformation-specific chemistry [26,27,28,29,30,31,32,33]. In this work, we have observed that the two main conformers of DMMP${}^{+}$ present in the experiment have distinct excited state dynamics while they behave very similarly in the ground state. The difference in the dynamics is governed by the different behavior of the excited states PES, which can channel vibrational energy in different ways for the two conformers. During internal conversion, the C${}_{s}$ conformer converts the electronic energy into vibrational energy along both P-O bonds symmetrically, while the C${}_{1}$ conformer goes through an asymmetric vibrational motion along the two P-O bonds. Ultimately, the difference in the dynamics leads to different dissociation products, as evidenced in the experimental fragmentation dynamics. Specifically, the slight difference in the phases of the oscillations in the fragments PO_2_C_2_H_7_^+^ and PO_2_CH_4_^+^ is evidence that they are initiated from different pathways.

The dynamics after the probe have been theoretically investigated here using static electronic structure calculations exploring the pathways, rather than dynamics. The most accurate theoretical study would require modeling of the dynamics after the probe excitation all the way to fragmentation to the two products. This process however is expected to take much longer than we are able to model with ab initio on the fly dynamics, especially since the fragmentation to PO_2_CH_4_^+^ is sequential [63]. The static calculations however, combined with the experimental observations of the different oscillation phases for the two fragments, provide the most plausible explanation for the underlying dynamics. Moreover, the ability to observe small (8%, or about 4 fs) phase shifts in the ion signals of different fragments demonstrates the potential power of pump-probe measurements to observe and possibly control nuclear dynamics in different conformers. Hence, pump-probe measurements can provide a complementary ultrafast spectroscopy tool to Coulomb explosion imaging [31] and electron diffraction [32,33] to study conformer-specific dynamics in molecules of biological interest.

## 5. Conclusions

We have investigated the dynamics of the DMMP cation after production by strong field adiabatic ionization. The pump-probe results using 800 nm probe show oscillations in the parent DMMP${}^{+}$ and fragments PO${}_{2}$C${}_{2}$H${}_{7}^{+}$ and PO${}_{2}$CH${}_{4}^{+}$ that were previously attributed to coherent oscillations of the P=O stretching bond [13]. Here we examined the details of the dynamics using trajectory surface hopping calculations. The TSH results demonstrate that indeed after vertical ionization to either the ground state D${}_{0}$ or the nearly isoenergetic first excited state D${}_{1}$, coherent oscillations along the P=O bond occur with a period of about 40 fs, very similar to the 45 fs observed experimentally. The probe pulse excites the cation to higher states D${}_{2}$ and D${}_{3}$ when it is resonant with the corresponding energy gap. Internal conversion from D${}_{2}$ or D${}_{3}$ can occur rapidly through conical intersections converting the electronic energy to vibrational energy further leading to dissociation.

The slight phase shift between the oscillations of the two fragment ions PO_2_C_2_H_7_^+^ and PO_2_CH_4_^+^ observed in the pump-probe measurements was attributed to the presence of two main conformers of DMMP at experimental conditions. These conformers exhibit very similar dynamics after ionization, both of them oscillating along D${}_{0}$ with a period of about 40 fs. Quite interestingly, however, the theoretical results show that the two conformers have very different dynamics after being excited by the probe pulse. They return to the ground state through different CoIns acquiring vibrational energy distributed differently among the internal degrees of freedom. Comparisons with the experimental observations indicate that the differences in the dynamics eventually lead to different fragments produced from the two conformers. Specifically, fragment PO_2_C_2_H_7_^+^ is expected to be produced from the C${}_{1}$ conformer, which has excess vibrational energy mainly along one of the P-O bonds, while PO_2_CH_4_^+^ can be produced sequentially after the production of PO_2_C_2_H_7_^+^ in the C${}_{s}$ conformer, which has both P-O bonds vibrationally excited after passing through CoIns. This combined experimental and theoretical study provides a unique example of conformer-specific dissociation dynamics, where conformers separated by very small energetic barriers can lead to different fragments, and indicates that these pump-probe measurements can provide a complementary ultrafast spectroscopy tool to study conformer-specific dynamics in molecules of biological interest.

## Data Availability

The data used for this study can be requested from the correspondence author.

## Supplementary Materials

The following supporting information can be downloaded at: https://www.mdpi.com/article/10.3390/molecules27072269/s1. Experimental information: mass spectra of DMMP ionized at 1300 nm pump with 400 nm and 800 nm probes. Theoretical information: geometries and energetics for all conformers; IPs at different geometries and levels of theory; orbitals; comparison of different levels of theory for pathways; details of conical intersections and the surface hopping dynamics; cartesian coordinates of stationary points.
