# Supplementary material for: Conformer-Specific Dissociation Dynamics in Dimethyl Methylphosphonate Radical Cation
Source: Molecules. 2022 Mar 31;27(7):2269. doi: 10.3390/molecules27072269 (PMC9000782; doi:10.3390/molecules27072269)
Supplement: Supplementary file 1 [file molecules-27-02269-s001.zip › molecules-1630240-SI.pdf]

Supplementary Material for: Conformer-Specific Dissociation Dynamics in Dimethyl Methylphosphonate Radical Cation

Vaibhav Singh,† Hugo A. López Peña,‡ Jacob M. Shusterman,‡ Patricia Vindel-Zandbergen,¶ Katharine Moore Tibbetts,‡ and Spiridoula Matsika*,†

†*Department of Chemistry, Temple University, Philadelphia, PA*

‡*Department of Chemistry, Virginia Commonwealth University, Richmond, VA*

¶*Department of Physics, Rutgers University at Newark, Newark, NJ*

E-mail: smatsika@temple.edu

The following information is available here. The mass spectra of DMMP ionized at 1300 nm pump with 400 nm and 800 nm probes can be found in Figure S1. The optimized geometries of neutral and cationic DMMP and four conformers of DMMP are shown in Figure S2 and Figure S3, respectively. The ionization potentials calculated at different neutral and cationic geometries of DMMP at the EOM-IP-CCSD/6-311+G(d) level of theory are listed in Table S1. Figures S4 and S5 comprise Dyson orbitals characterizing the holes of the first 5 IP states at symmetric and asymmetric neutral geometries, respectively. Figures S6 and S7 contain orbitals involved in SA7-CASSCF(13,9) calculations for symmetric and asymmetric conformers, respectively. Section 6 includes LIICs connecting the various geometries at different levels of theories. Figure S13 shows the branching vectors of D_2/D_3 conical intersections. The distributions of P-O bond lengths of the geometries generated with the Wigner distribution are shown in Figure S15. The other results of surface hopping can also be found in Section 9, such as energies of D_0 and D_1 states vs. time, population dynamics on D_0 and D_1 states, P=O dynamics, etc. The comparison between the dynamics of symmetric vs. asymmetric conformers is also made with the help of Figure S20. The cartesian coordinates of different stationary points relevant to this study can be found in Section 10.

1 Experiment

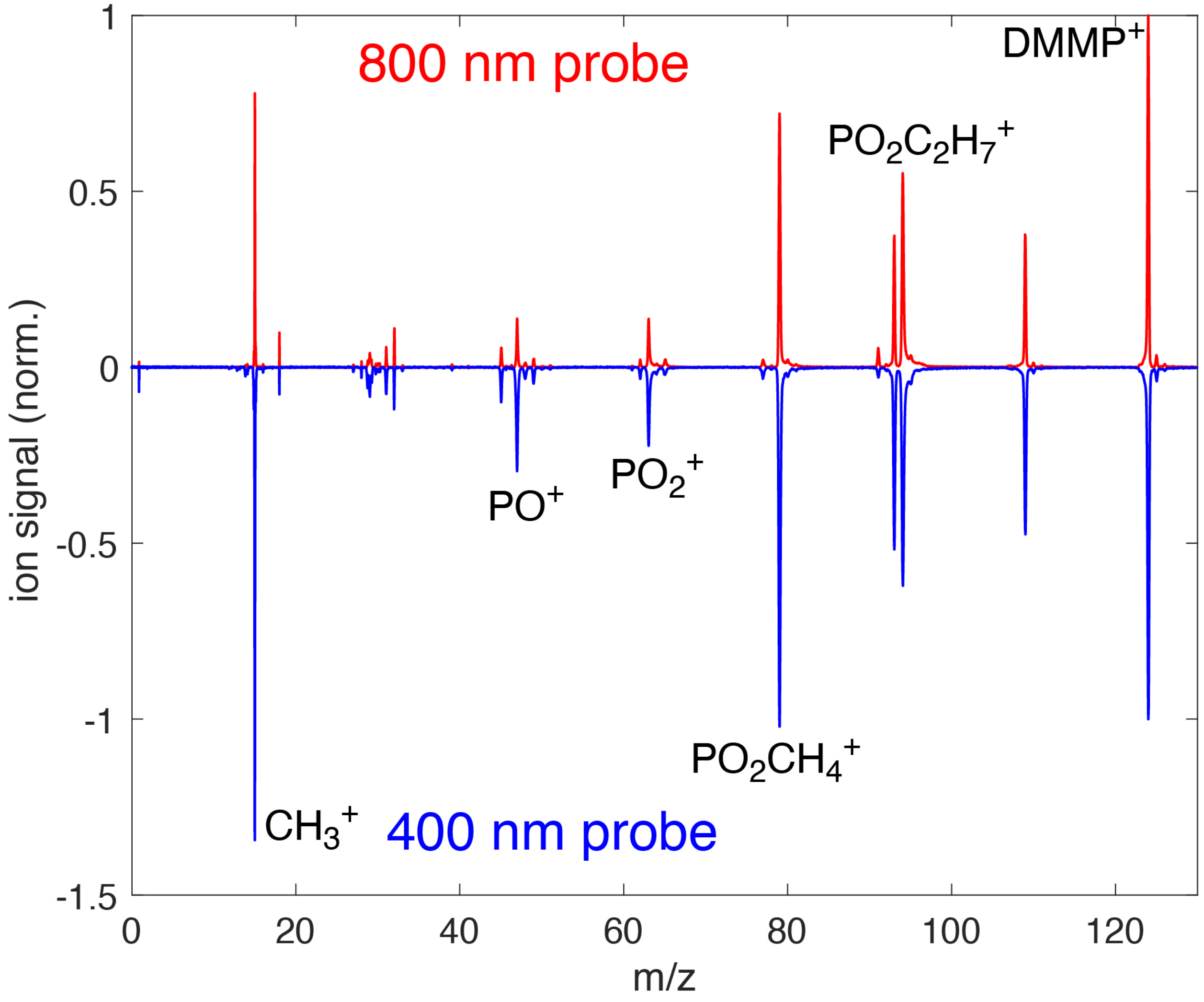

Figure S1. Mass spectra of DMMP ionized at 1300 nm with 800 nm (red) and 400 nm (blue) probe pulses at a delay of +800 fs. Signals are normalized to the yield of the parent ion DMMP+ and significant fragments are labeled.

2 Optimized Geometries

Figure S2 shows the optimized geometries of the asymmetric (C_1) and the symmetric (C_s) con-formers at the neutral ground state minimum S_0 and at the cationic ground state minimum D_0 at the B3LYP/6-311+G(d) level of theory. The two additional low energy conformers of

the neutral DMMP and their relative populations are included in Fig. S3. The C_1 and the C_s conformers (1 and 2) are very close in energy both in the neutral and cationic molecule, while conformers 3 and 4 are higher in energy and they barely contribute to the total population of the neutral DMMP at the experimental conditions. Then, just the C_1 and C_s conformers are considered in the calculations.

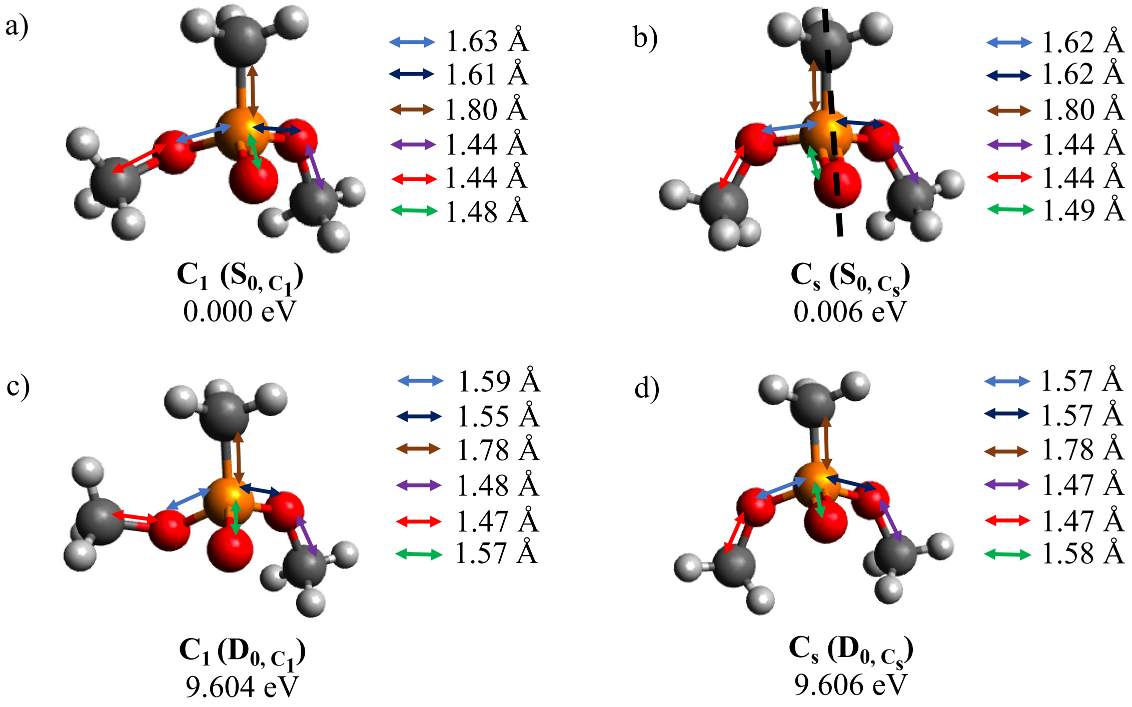

Figure S2: Optimized geometries of the C_1 and the C_s conformers at the neutral ground state minimum S_0 (a and b) and at the cationic ground state minimum D_0 (c and d). The black dashed line represents the mirror plane in the C_s conformer and contains the C-P and P=O bonds. The coloured arrows represent the different bond lengths. The energies are given with respect to the S_{0,C_1} ground state minimum.

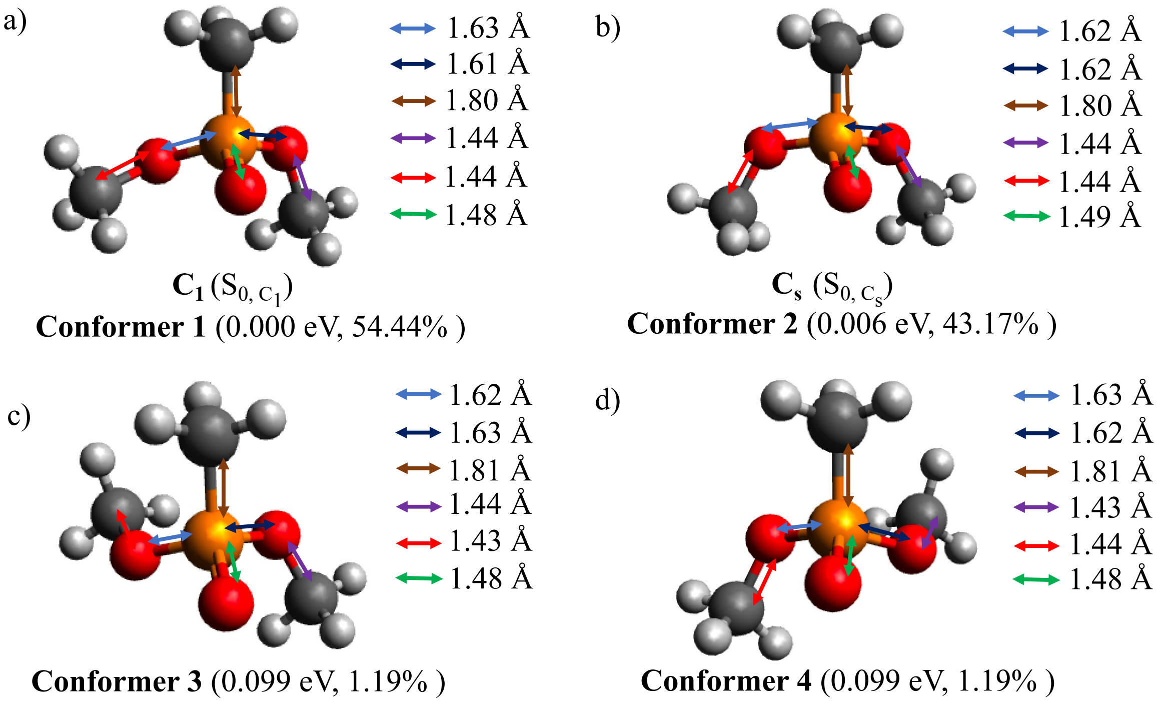

Figure S3. Optimized geometries of the low energy conformers of neutral DMMP. For each conformer, we show the bond distances with the colored arrows representing the different bonds. The values in brackets are their relative energies with respect to the S_{0,C_1} geometry and and their relative populations at the experimental conditions, according to a Boltzmann distribution.

3 Ionization Potentials

The vertical ionization potentials (IPs) calculated at EOM-IP-CCSD/6-311+G(d) level for the C_1 neutral (S_{0,C_1}) and C_s neutral (S_{0,C_s}) geometries are given in Table S1 and plotted in Figure 3. In addition, we performed EOM-IP-CCSD calculations with a larger basis set, cc-pVTZ, to check the accuracy of these energies (Table S2). The difference of the predicted

energies using different basis set is not significant, and hence the 6-311+G(d) basis was used in subsequent calculations.

Table S1. Ionization Potentials calculated at the S_{0,C_1}, S_{0,C_s}, D_{0,C_1} and D_{0,C_s} geometries using EOM-IP-CCSD/6-311+G(d). The energy values are given in eV with respect to the gr

	S_{0,C_1}	S_{0,C_s}	D_{0,C_1}	D_{0,C_s}
**D_0**	10.402	10.238	9.600	9.578
**D_1**	10.495	10.454	10.098	9.956
**D_2**	10.999	10.890	11.715	11.651
**D_3**	11.579	11.563	12.548	12.251
**D_4**	12.118	12.061	13.098	13.378

Table S2. Ionization Potentials (in eV) calculated at EOM-IP-CCSD/6-311+G(d) and EOM-IP-CCSD/cc-pVTZ levels of theory for S_{0,C_1} and S_{0,C_s} ge-ometries. ΔE and ΔE' are the differences between the energies calculated with the two different basis sets at S_{0,C_1} and S_{0,C_s}, respectively.

	S_{0,C_1}			S_{0,C_s}		
	**6-311+G(d)**	**cc-pVTZ**	**ΔE**	**6-311+G(d)**	**cc-pVTZ**	**ΔE'**
**D_0**	10.313	10.434	0.121	10.238	10.373	0.135
**D_1**	10.406	10.522	0.116	10.454	10.556	0.102
**D_2**	10.911	11.068	0.157	10.890	11.042	0.152
**D_3**	11.491	11.647	0.156	11.563	11.692	0.129
**D_4**	12.029	12.138	0.109	12.061	12.161	0.100

4 Excited states of $DMMP^+$: SA-CASSCF(13,9) and EOM-IP-CCSD energies

Table S3 compares the excited state energies of the DMMP (C_1 conformer) cation obtained from different states averaged CASSCF(13,9)/6-311+G(d), CASSCF(13,9)/6-311G(d) and EOM-IP-CCSD/6-311+G(d). The calculated energies are very sensitive to the level of theory, and CASSCF calculations overestimate the excited state energies compared to EOM-IP-CCSD.

Table S3. States averaged CASSCF(13,9) and EOM-IP-CCSD energies at S_{0,C_1} geometry. In all the rows without a basis set, 6-311+G(d) was used.

	D_0	D_1	D_2	D_3	D_4
**7SA-CAS(13,9)**	0	0.115	1.295	1.907	2.210
**6SA-CAS(13,9)**	0	0.120	1.300	1.917	2.310
**4SA-CAS(13,9)**	0	0.443	1.309	2.231	
**3SA-CAS(13,9)**	0	0.164	2.449		
**2SA-CAS(13,9)**	0	0.158			
**2SA-CAS(13,9)/6-311G(d)**	0	0.612			
**5SA-CAS(13,9)/6-311G(d)**	0	0.054	1.594	2.172	2.549
**EOM-IP-CCSD**	0	0.093	0.598	1.178	1.716

5 Molecular Orbitals

Figures S4 and S5 show the Dyson orbitals of the $DMMP^+$ states at S_{0,C_1} and S_{0,C_s} geometries. One can clearly see that D_0 and D_1 in both conformers have the same character, originated by the ionization of an electron in orbitals located along the P=O bond. These lead to very similar dynamics of the two lowest cationic states in the C_1 and C_s conformers. On the other hand, the different character of D_2 and D_3 states can be responsible of the different fragments observed experimentally. The 9 orbitals (P=O π orbital and the lone pairs of the oxygen molecules) included in the active space in the state averaged CASSCF calculations are also shown in Figs. S6 and S7 at the S_{0,C_1} and S_{0,C_s} geometries, respectively.

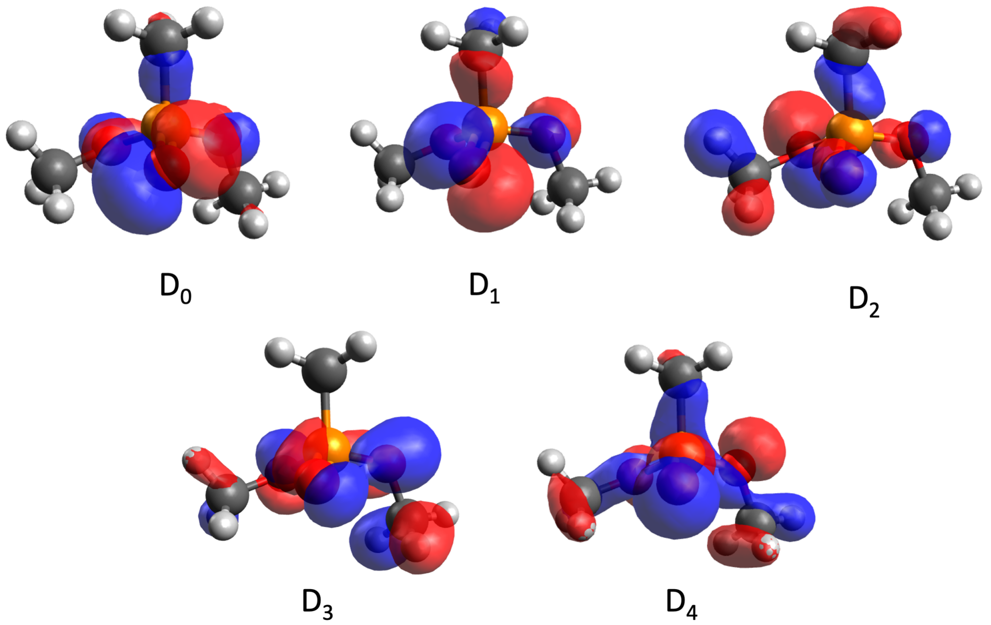

Figure S4. Dyson orbitals characterizing the hole of the first five cationic states at S_{0,C_1} geometry obtained from EOM-IP-CCSD/6- 311+G(d) calculations

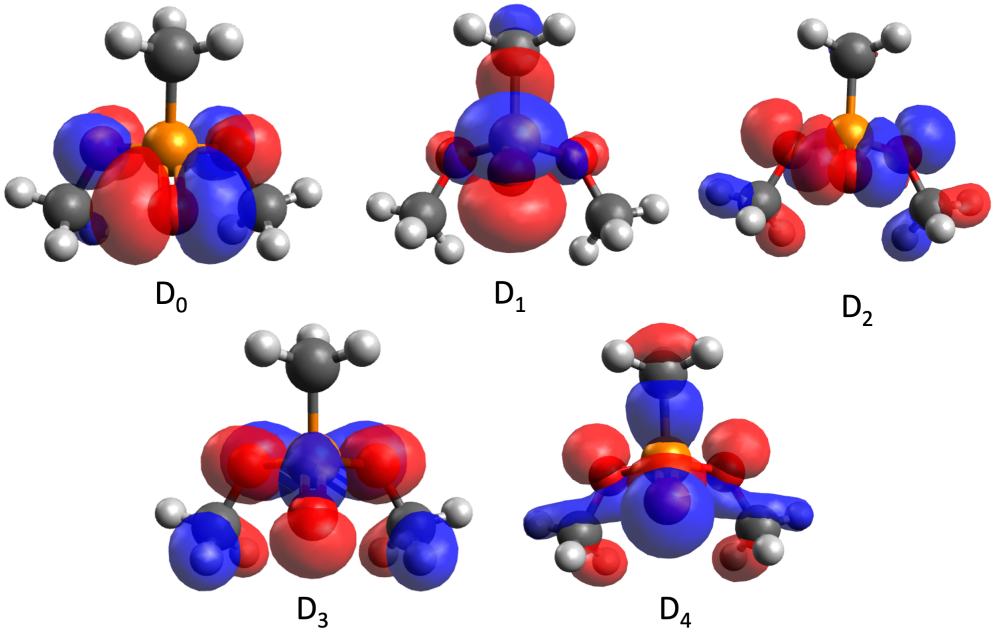

Figure S5. Dyson orbitals characterizing the hole of the first five cationic states at S_{0,C_s} geometry obtained from EOM-IP-CCSD/6- 311+G(d) calculations

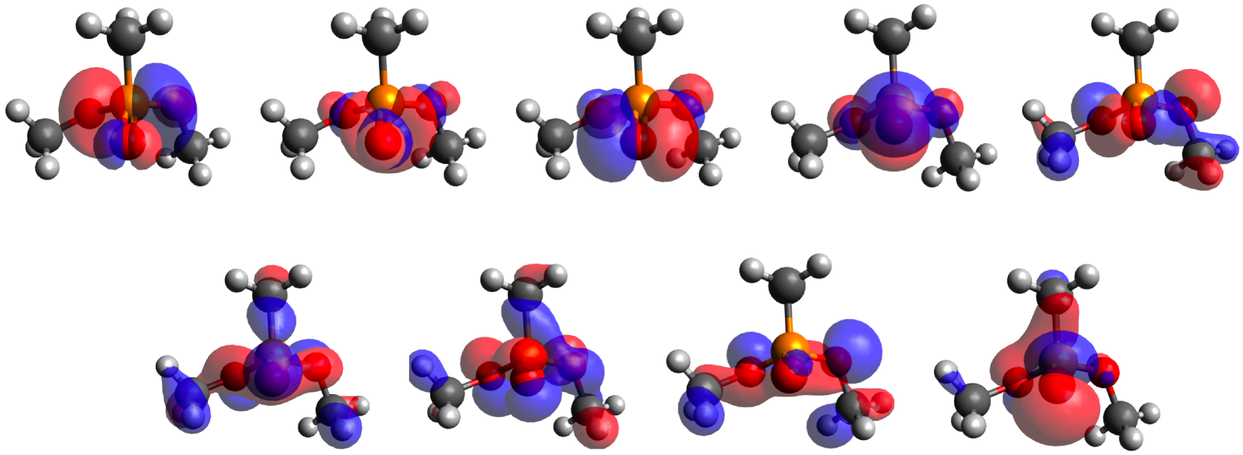

Figure S6. Orbitals included in the active space of SA7-CASSCF(13,9) calculations for S_{0,C_1} geometry.

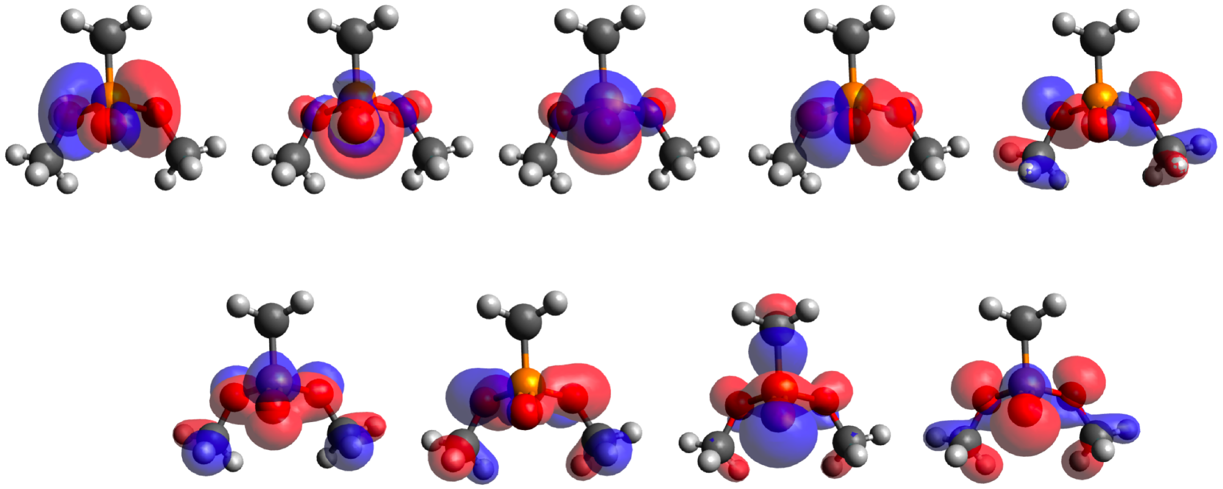

Figure S7. Orbitals included in the active space of SA7-CASSCF(13,9) calculations for S_{0,C_s} geometry.

6 Linear Interpolations

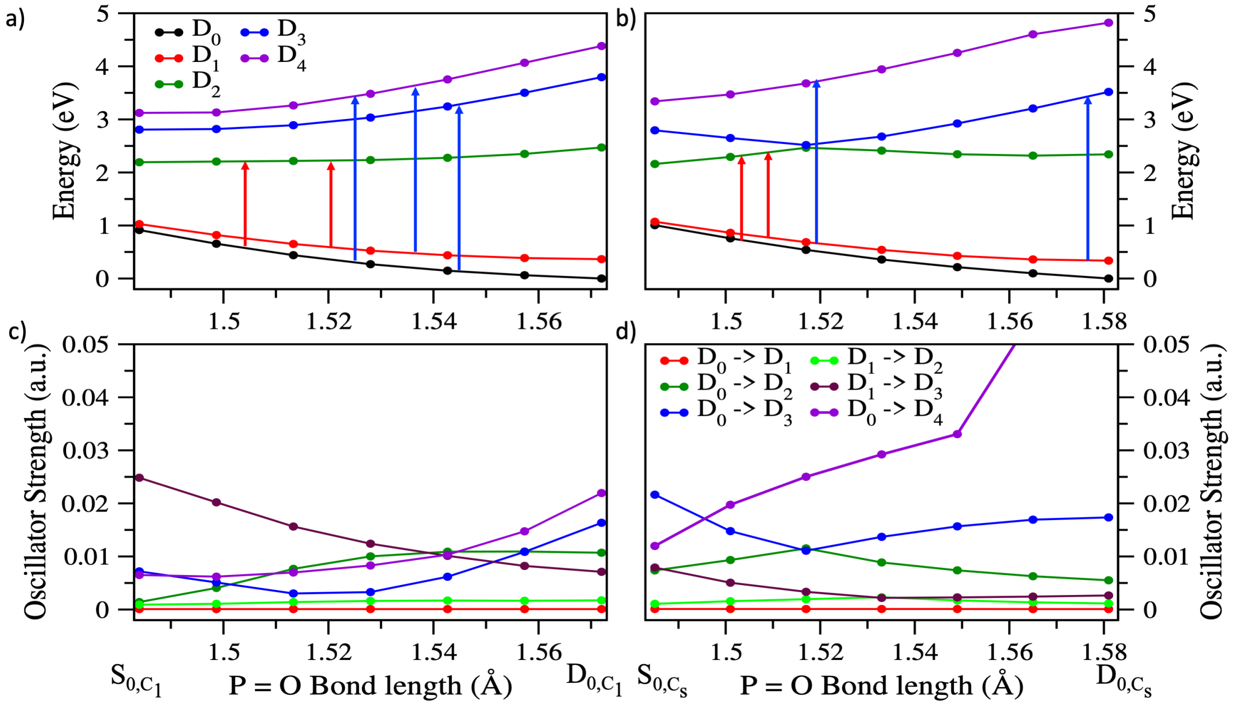

Figure S8. Energies along LIIC paths connecting vertical ionization energies from S_0 equilibrium geometry to the minimum of the cation D_0 for a) the C_1 conformer and b) the C_s conformer. The transitions due to probe light of 400 nm (3.1 eV) and 800 nm (1.6 eV) are represented with blue and red arrows respectively. c) and d) Oscillator strengths along the paths shown in a and b, respectively. All the calculations were done at SA7-CASSCF(13,9)/6-311+G(d) level of theory.

Figures S8 and S9 show the excited state energies and oscillator strengths of the transitions from the ground state (D_0) to the four excited states of $DMMP^+$ were calculated along the LIIC paths connecting the Franck Condon region (S_0 equilibirum geometry) and the D_0 minimum at SA7-CASSCF(13,9)/6-311+G* and EOM-EE-CCSD/6-311+G(d) levels of theory. Comparing to EOM-IP-CCSD calculations, that give the most accurate energies, EOM-EE-CCSD energies do not deviate much from those values, while SA-CASSCF overestimates the energies of the higher states. This is evident in Fig.S10 that compares the results obtained from CASSCF and EOM-IP-CCSD methods by superimposing the energies along the LIIC paths for the C_s and C_1 conformers.

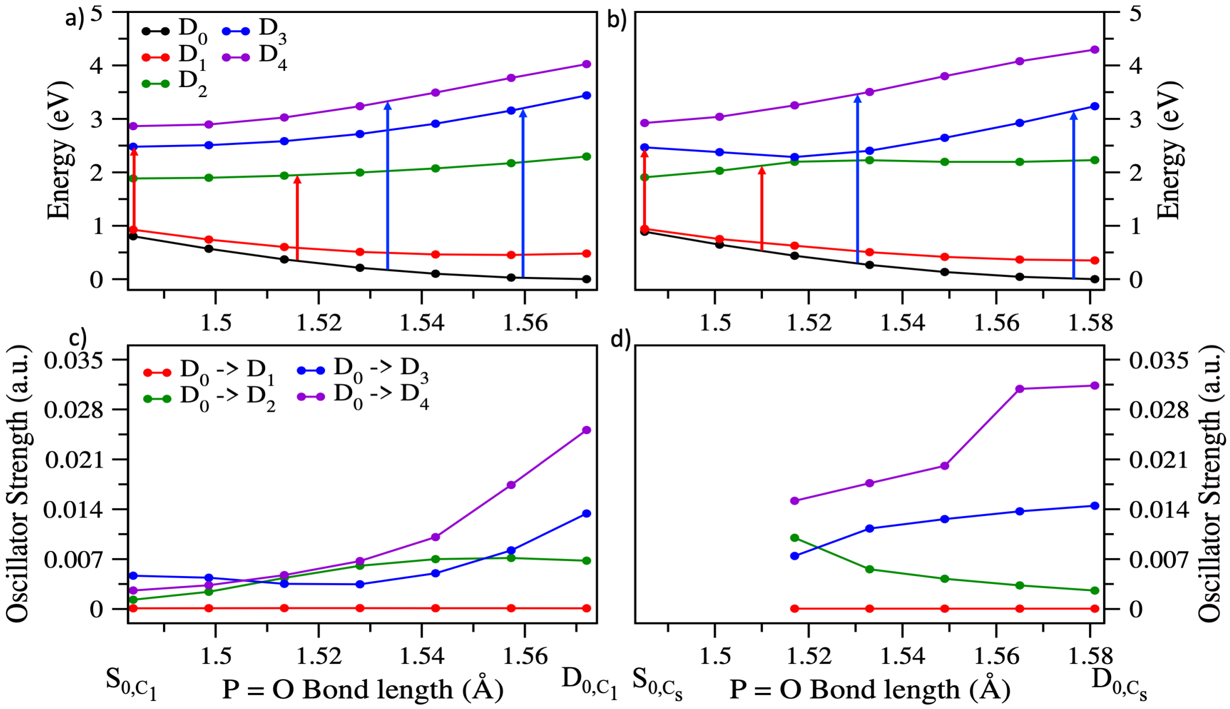

Figure S9. Same as Fig.S8 at EOM-EE-CCSD/6-311+G(d) level of theory. The oscillator strengths calculated at the first two points of 'd' are ambiguous and hence are omitted. The ambiguity might have occurred due to the mixing of characters of the states D_0 and D_1 at the first two points where they are nearly degenerate.

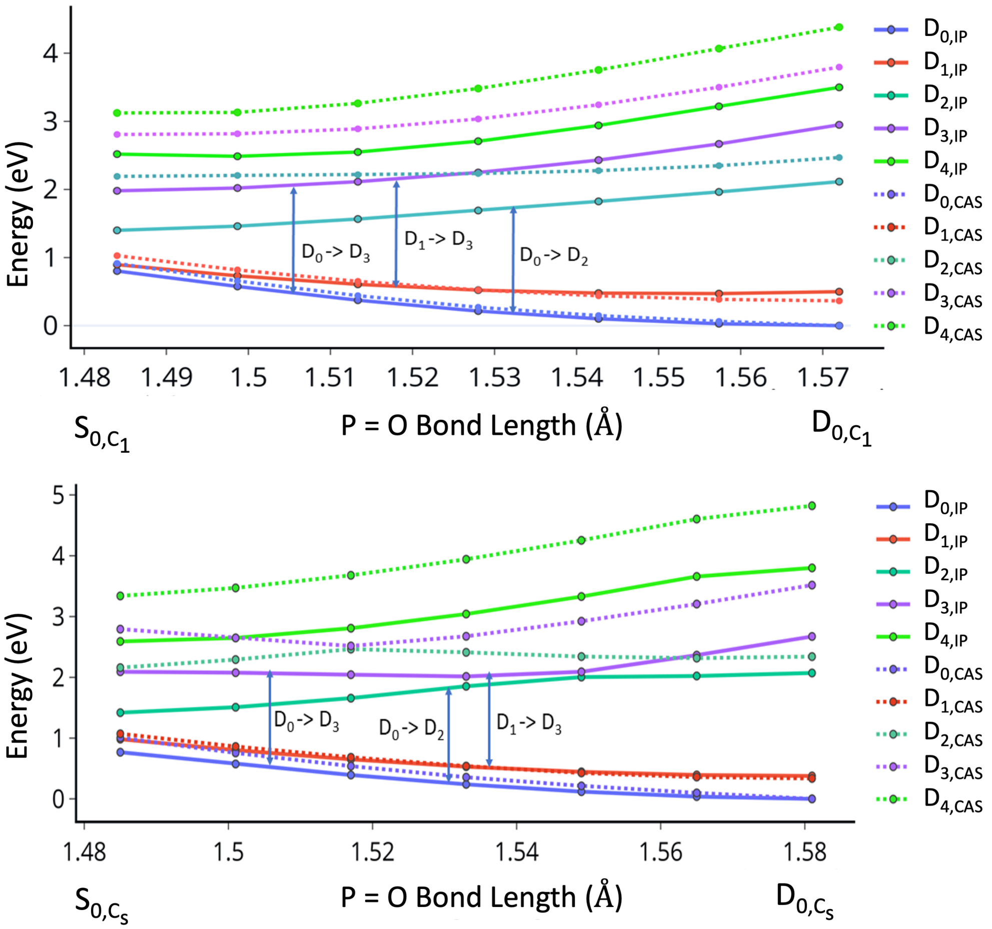

Figure S10. Energies calculated along LIIC path from C_1 S_0 geometry (S_{0,C_1}) to C_1 D_0 geometry (D_{0,C_1}) (top) and from C_s S_0 geometry (S_{0,C_s}) to C_s D_0 geometry (S_{0,C_s}) (bottom) at EOM-IP-CCSD/6-311+G(d) (solid lines) and at SA7-CASSCF(13,9)/6-311+G(d) (dashed lines) levels of theory.

6.1 EOM-EE-CCSD using ROHF vs UHF

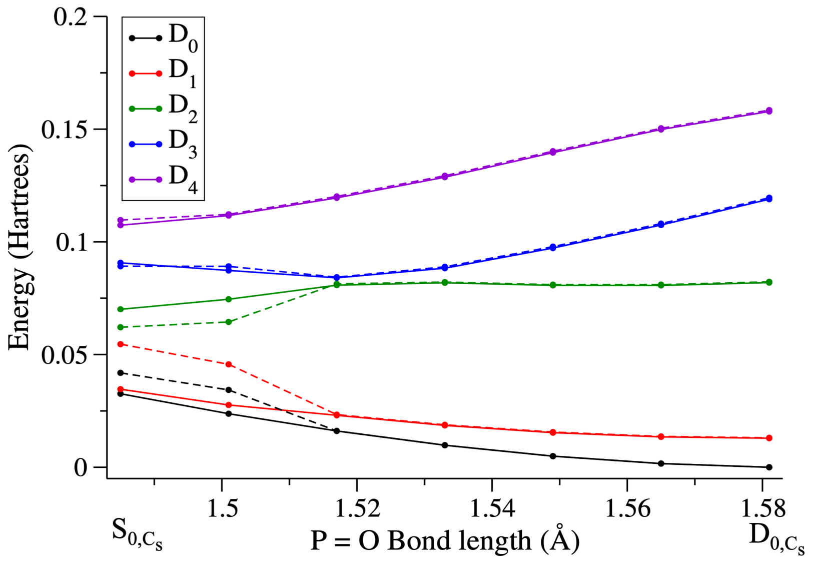

Figure S11. LIIC connecting the C_s neutral (S_{0,C_s}) to the C_s cationic (D_{0,C_s}) geometries. The calculations are done at EOM-EE-CCSD/6-311+G(d) level of theory using two different sets of Hartree Fock orbitals as initial guess, one optimized with ROHF (solid line) and another with UHF (dashed line) methods.

Fig. S11 shows the energies of electronic states calculated along the LIIC paths at the EOM-EE-CCSD/6-311+G(d) level of theory with 'Unrestricted Hartree Fock' (UHF) and 'Restricted Open shell HF' (ROHF) orbitals as the initial guesses. One can see that at the geometries near S_{0,C_s}, UHF orbitals do not converge to correct results. Generally, UHF orbitals are used by default for open-shell EOM-EE-CCSD calculations in Q-Chem packages. The calculations started with ROHF orbitals produce smoother curves and, the energies overlap with UHF energies along the LIICs except at the geometries near the vertical ionization geometries. This is probably due to the degeneracy of D_0 and D_1 states near the S_{0,C_s} geometry.

6.2 Connecting C_s to C_1 minima

Since the two cationic conformers have about equal energies, we explored a possible conversion pathway between them. Fig. S12 shows the transition state connecting the C_s (D_{0,C_s}) and C_1 (D_{0,C_1}) geometries. While the barrier is small, only 0.14 eV, we will show that we did not observe conversion between these two minima in trajectory surface hopping dynamics.

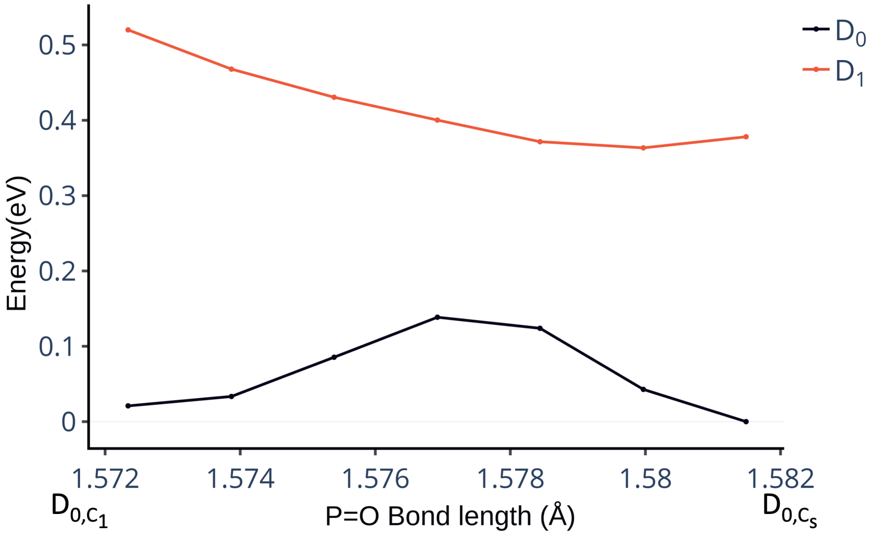

Figure S12. Energies of the first two electronic states of $DMMP^+$ calculated along LIIC from C_S D_0 geometry (D_{0,C_s}) to C_1 D_0 geometry (D_{0,C_1}). The calculations were done at the EOM-IP-CCSD/6-311+G(d) level of theory.

7 Conical intersections

The two branching vectors around CoIns are defined as the difference of the gradients of the two states, and the coupling between them. They indicate the main vibrational motions that are activated when a molecule goes through a CoIn. The branching vectors for the D_2/D_3 CoIns located for $DMMP^+$ are shown in Fig. S13, and they highlight that the PO bonds are primarily activated.

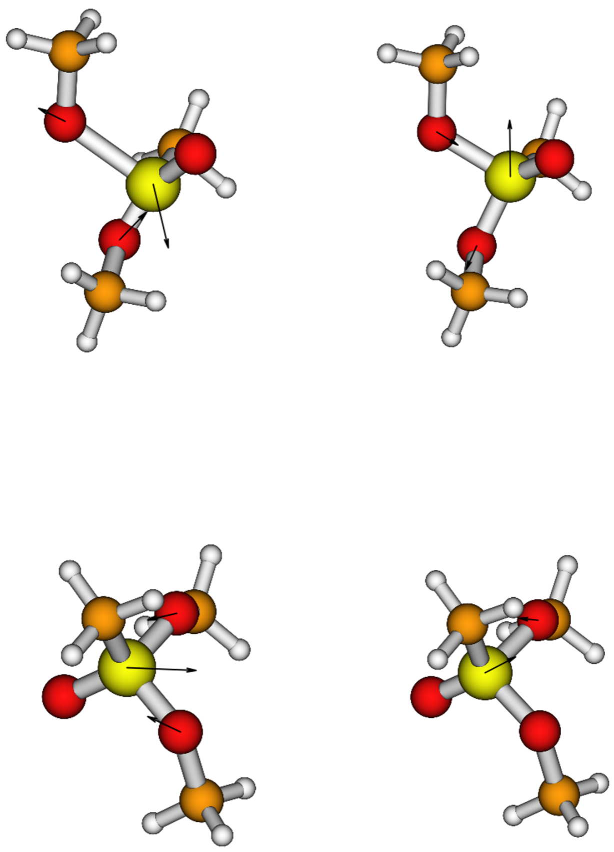

(a)

(b)

Figure S13. Branching vectors of the D_2/D_3 conical intersection in (a) the C_1 conformer, and (b) the C_s conformer.

8 Hydrogen transfer

Fig. S14 shows the pathway connecting the transition state for hydrogen migration to the final HT isomer. It is clear from this plot that after the transition state the path is practically downhill stabilizing the energy by more than 1.5 eV. The small barrier at the second step is because the TS_{HT} is optimized at a different level of theory than the LIIC path. So it is rather technical rather than a real extra barrier.

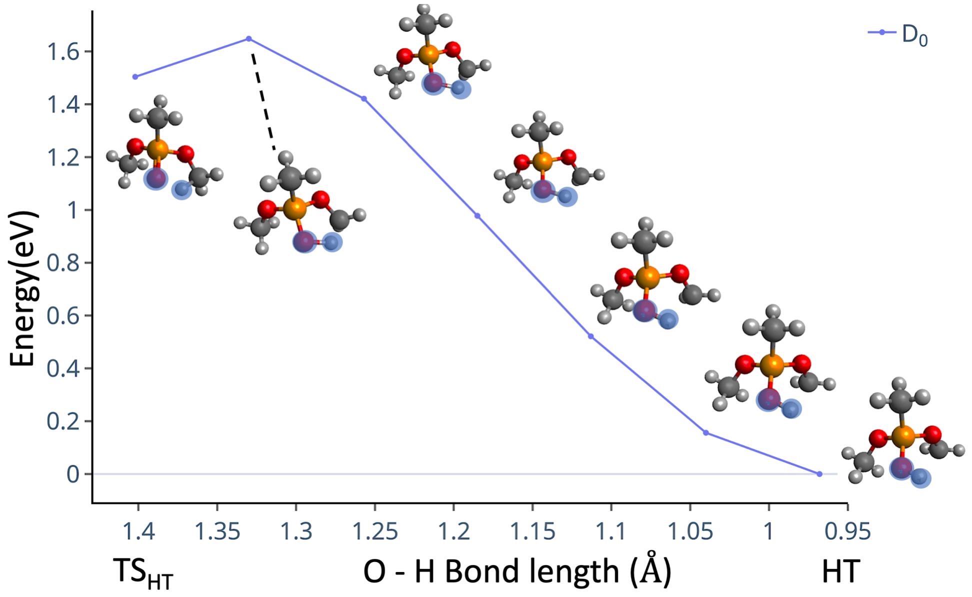

Figure S14. D_0 plotted along LIIC from TS_{HT} to HT vs O-H bond length. The O and H atoms used for the x-axis are highlighted. The calculations were done at EOM-EE-CCSD/6-311+G(d) level of theory.

9 Trajectory Surface Hopping

Surface hopping dynamics calculations were done to study the dynamics of the C_1 conformer. In Fig. S15 we plotted histograms of the P-O bond lengths of the 100 geometries obtained from the Wigner distribution.

The mean values and individual energies of the states D_0 and D_1 for the dynamics starting in the D_0 (Dyn_D_0) and D_1 (Dyn_D_1) states are plotted in Fig. S16. The populations

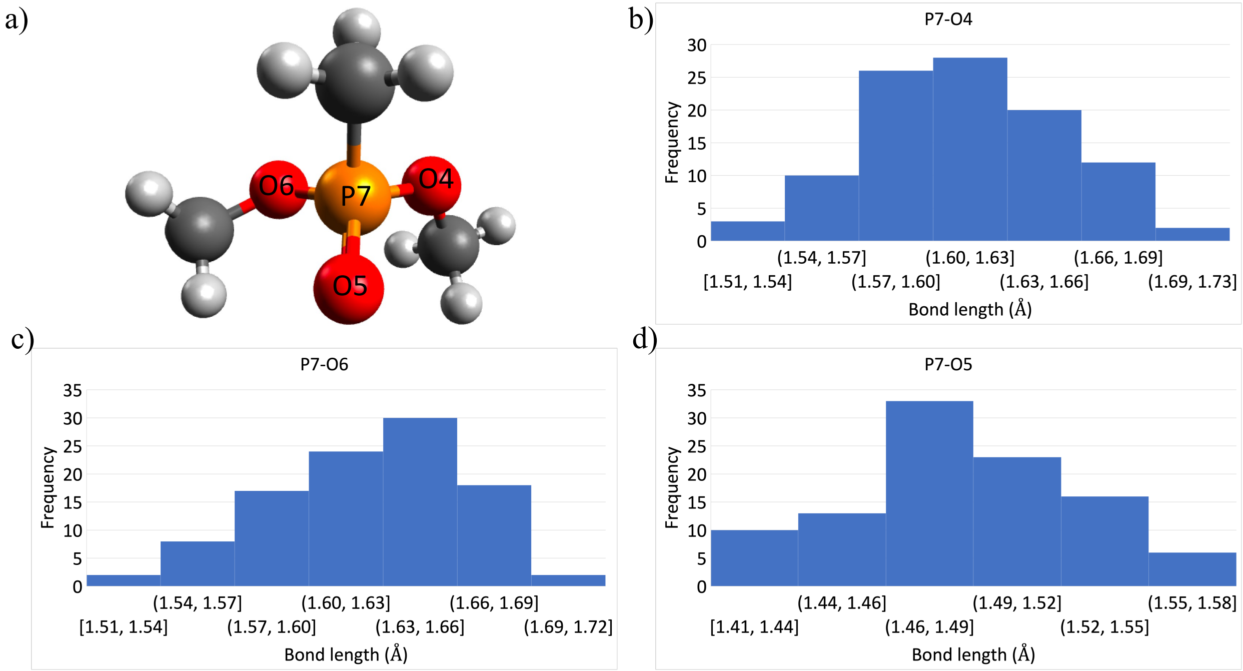

Figure S15. The distributions of 3 P-O bond lengths of the 100 initial conditions produced with the harmonic oscillator Wigner distribution. For the numbering of the atoms, please refer to 'a)' in the figure.

dynamics is plotted in Figure S17. When the initial population is on D_0 there is very small population transfer to D_1. When the initial population is on D_1 most of it transfers to the ground state D_0 but about 30% remains on D_1 after 200 fs. Nevertheless, regardless of whether population is on D_0 or D_1 the motion of the molecule does not change. This is shown in Fig. S18 which shows the oscillations of the the P=O bond.

Another interesting effect we observed in the dynamics is that despite the barrier between the symmetric and asymmetric minimum is very low, there is no interconversion between the two conformers. This is clearly evident in Fig. S19 which shows the variation of mean values of the dihedral angles D1 and D2 (as defined in the figure) along the dynamics. In the C_1 system the two dihedrals are different, while in the C_s molecule D1 and D2 should be the same. We show that during the dynamics the dihedrals remain different at all times. Therefore, we can conclude that the C_1 conformer never converts into C_s conformer during the dynamics.

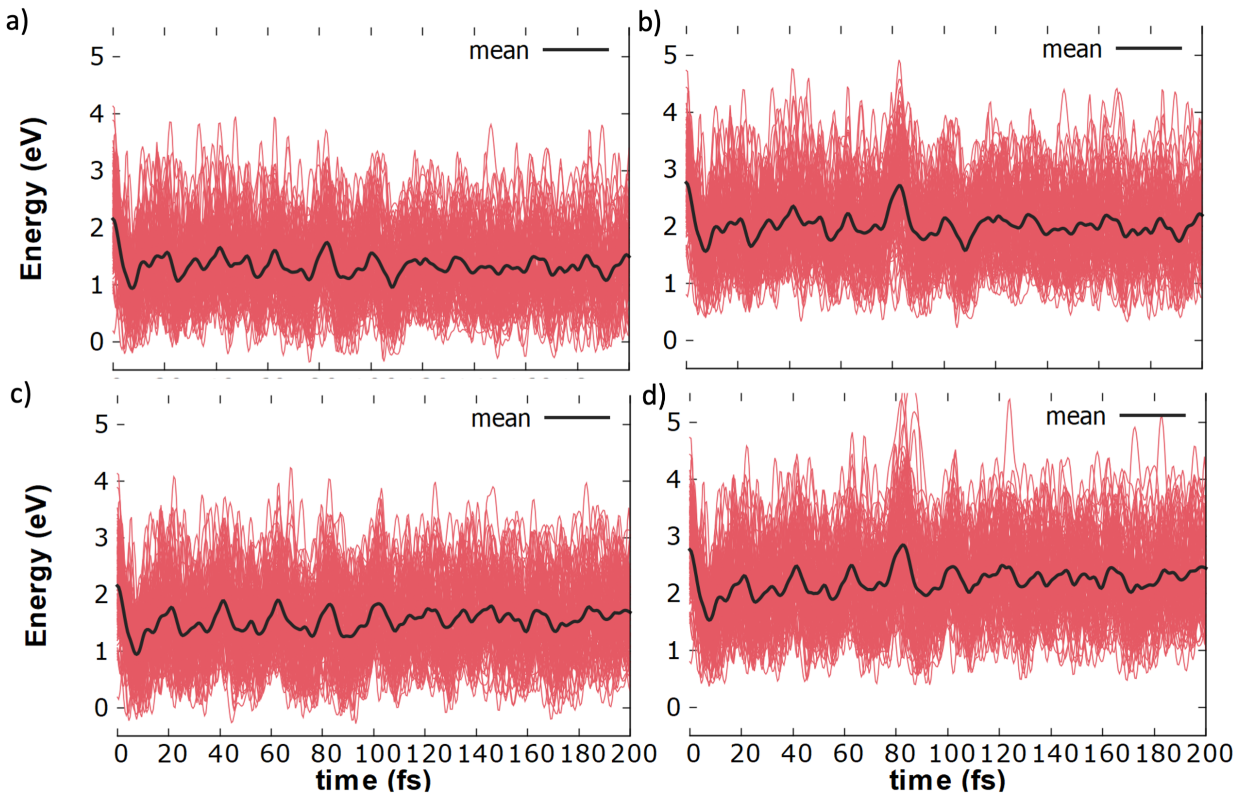

Figure S16. a) and b) Energies of the D_0 and D_1 states for the dynamics starting on D_0 (Dyn_D_0). c) and d)Energies of the D_0 and D_1 states for the dynamics starting on D_1 (Dyn_D_1). Black thick lines represent the mean values, while red thin lines correspond to individual trajectories

Figure S20 compares P=O bond lengths and D_0 and D_1 energies for one trajectory in the C_s and C_1 conformers. These single trajectories correspond to dynamics started from the equilibrium geometries and with zero velocities. One can clearly see that both conformers show very similar behavior for dynamics starting in D_0 and D_1. The bond lengths and D_0,D_1 energies for the different conformers oscillate with the same period, demonstrating that the ground state dynamics is the same for the C_s and C_1 conformers.

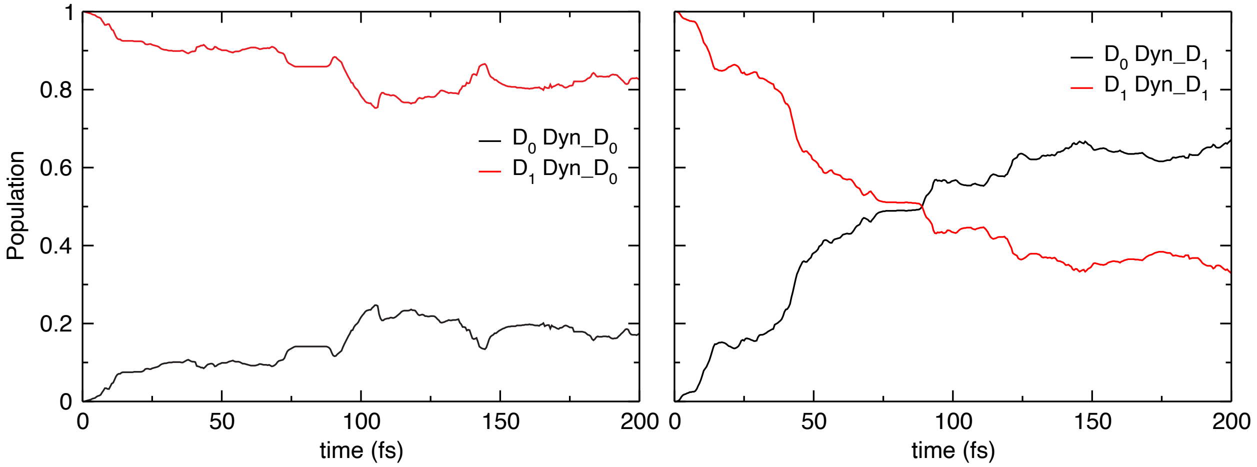

Figure S17. Population dynamics of the first two cationic states of the C_1 conformer starting with D_0 as initial state (left) and starting in D_1 (right)

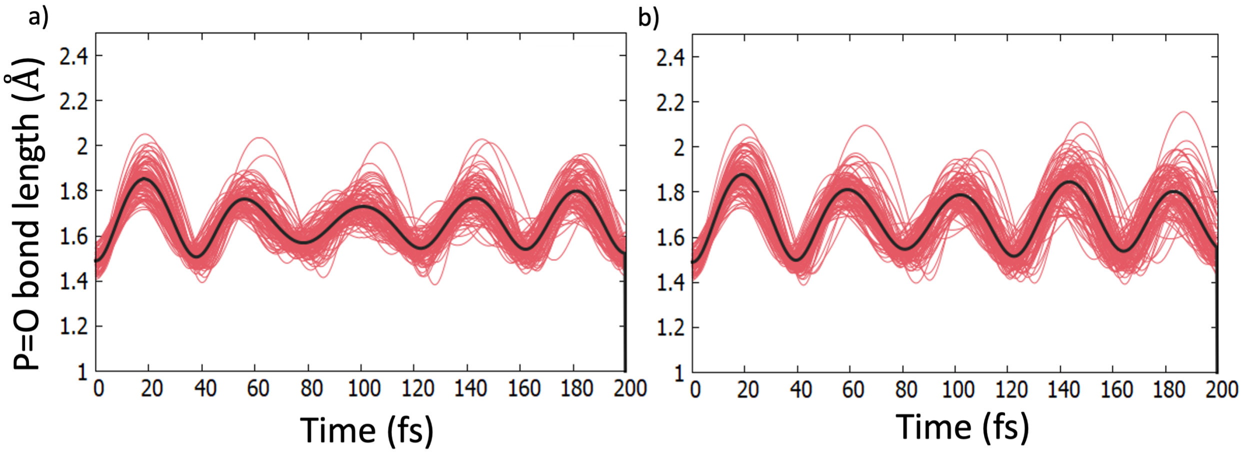

Figure S18. Time variation of the P=O bond lengths for dynamics staring in D_0 state (left) and in D_1 state (right). Black thick lines represent the mean values, while red thin lines correspond to individual trajectories.

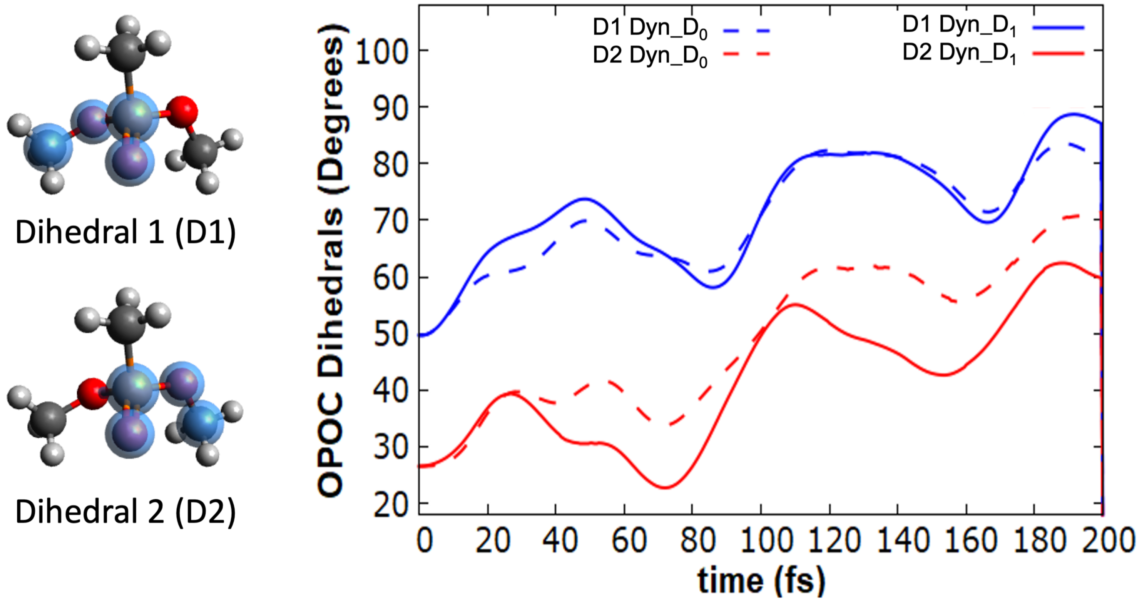

Figure S19. Time variation of the mean values of dihedral angles D1 (blue) and D2(red) for the dynamics of the asymmetric molecule starting on D_0 (dashed lines) and D_1 (solid lines).

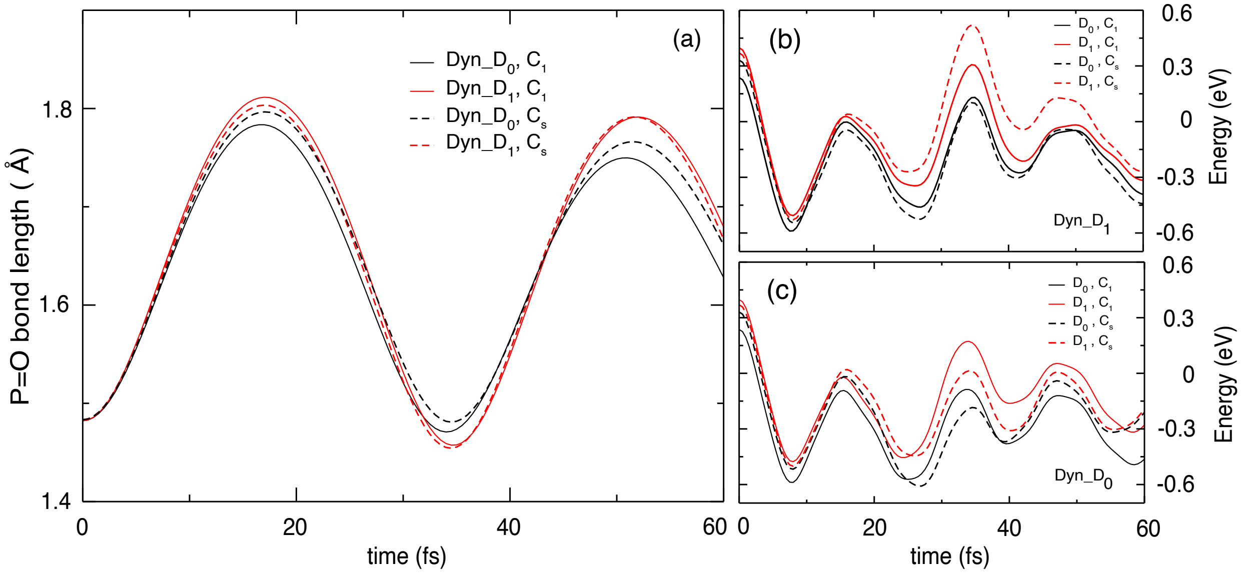

Figure S20. Comparison of the dynamics for one trajectory between the C_s and C_1 conformers starting in D_0 (Dyn_D_0) and in D_1 (Dyn_D_1) states. (a) P=O bond lengths and the D_0 and D_1 energies for (b) Dyn_D_1 and (c) Dyn_D_0 dynamics.

10 Cartesian Coordinates of the stationary points

C_1 neutral geometry, S_{0,C_1} (Conformer 1)

C 0.0000000000 0.0000000000 0.0000000000

H 0.0000000000 0.0000000000 1.0881700000

O 1.3812958431 0.0000000000 -0.4013759567

H -0.5076646667 0.8917348542 -0.3715708256

H -0.5003911316 -0.8979581645 -0.3682564510

P 1.7878283785 0.1511304000 -1.9529886300

O 1.1744227547 1.2769659016 -2.7009814105

O 1.3788209271 -1.3099201905 -2.5401705533

C 0.9974838372 -1.4825948352 -3.9154843368

C 3.5874613519 0.1553052710 -1.8283317643

H 3.9119418272 1.0641996314 -1.3201273105

H 4.0139734624 0.1456796296 -2.8328305260

H 3.9384024722 -0.7161787159 -1.2753946905

H 0.4468258728 -2.4205688355 -3.9670790373

H 1.8855447485 -1.5489335976 -4.5496831580

H 0.3693613084 -0.6566522983 -4.2496139957

C_s neutral geometry, S_{0,C_s} (Conformer 2)

P -0.0001619722 -0.4300157047 0.1699136614

C -1.9947335789 1.3226529757 -0.1485979789

C -0.0002610724 -2.1422242528 -0.3825776216

C 1.9956706941 1.3214782195 -0.1486720041

O 1.2963833549 0.1540259325 -0.6104945272

O 0.0006702310 -0.1514346142 1.6290477963

O -1.2977179494 0.1533693987 -0.6091577618

H -2.9897538672 1.2815257864 -0.5886204131

H -2.0649618820 1.3268948505 0.9397803627

H -1.4856926052 2.2271090957 -0.4909765830

H -0.8889605946 -2.6418244141 0.0040717902

H 0.8897616793 -2.6411944355 0.0018635247

H -0.0015938982 -2.1921782884 -1.4716917963

H 2.9894209532 1.2806281912 -0.5915808662

H 2.0689080186 1.3227831101 0.9395154862

H 1.4865604311 2.2273642840 -0.4871448578

Conformer 3

C -0.0960200000 0.2918680000 0.1117360000

H -0.1333940000 0.2788050000 1.1994420000

O 1.2368010000 -0.1184390000 -0.2493040000

H -0.2998820000 1.2958490000 -0.2613590000

H -0.8355770000 -0.4041210000 -0.2910720000

P 1.7339290000 -0.0182670000 -1.7927500000

O 1.1750360000 1.1080050000 -2.5689550000

O 1.3219580000 -1.4398300000 -2.4570710000

C 1.6027910000 -2.7089060000 -1.8537050000

C 3.5279150000 -0.0459070000 -1.5783050000

H 3.8411560000 0.8885490000 -1.1116280000

H 3.9996400000 -0.1250240000 -2.5590300000

H 3.8503040000 -0.8776330000 -0.9502160000

H 2.6664450000 -2.9504000000 -1.9332560000

H 1.0279720000 -3.4477370000 -2.4095340000

H 1.3002360000 -2.7224710000 -0.8042530000

Conformer 4

C 1.7734510000 -0.6633030000 0.9296680000

H 1.2512370000 -0.3840800000 1.8431880000

O 1.4474710000 0.3358060000 -0.0445180000

H 2.8494440000 -0.6802410000 1.1244390000

H 1.4428360000 -1.6527650000 0.6059510000

P 1.7748300000 0.1884790000 -1.6266490000

O 1.1867590000 1.3067780000 -2.3927150000

O 1.2397270000 -1.2920190000 -2.0280900000

C -0.1218320000 -1.4789470000 -2.4596180000

C 3.5614210000 -0.0422600000 -1.7685960000

H 4.0636040000 0.8491200000 -1.3892580000

H 3.8172340000 -0.1688790000 -2.8210420000

H 3.9047230000 -0.9177570000 -1.2156350000

H -0.3641080000 -0.7871510000 -3.2667680000

H -0.1905090000 -2.5069460000 -2.8105660000

H -0.8138390000 -1.3260780000 -1.6281340000

C_1 cationic geometry (D_{0,C_1})

C 0.0000000000 0.0000000000 0.0000000000

H 0.0000000000 0.0000000000 1.0846700000

O 1.4278997544 0.0000000000 -0.3737355900

H -0.4772555810 0.8954148168 -0.3931305151

H -0.4560757197 -0.9048038573 -0.3943383269

P 2.0527928891 0.2922933430 -1.7631418425

O 1.5571579184 1.6341529913 -2.4158375877

O 1.4259513403 -0.7239204889 -2.8104193247

C 1.8598742036 -1.1353084435 -4.1473367908

C 3.8186722525 0.1812358818 -1.5640855815

H 4.1378116159 0.9272515947 -0.8321911384

H 4.3178986127 0.3850350774 -2.5126653652

H 4.0895355567 -0.8102097440 -1.1949760530

H 1.3652147688 -2.0885738335 -4.3053637530

H 2.9427875795 -1.2400928546 -4.1796206877

H 1.5036966516 -0.3971045096 -4.8633662774

C_s cationic geometry ($\mathbf{D_{0,C_s}}$)

P 0.0000000000 0.0000000000 0.0000000000

C 0.0000000000 0.0000000000 2.7664900000

C 1.3552153838 0.0000000000 -1.1465377386

C -2.2247462914 -1.6495197055 0.1384671076

O -0.9402608088 -1.1895519619 -0.4061476565

O -0.9470334594 1.2661593219 -0.0328453694

O 0.6213949731 -0.0550684371 1.4351372209

H 0.7102113882 0.5295559513 3.3933721671

H -0.9485720529 0.5310940887 2.7207737276

H -0.1263728285 -1.0256691330 3.1075802598

H 2.0005009911 0.8547094290 -0.9356530377

H 0.9631866165 0.0779606796 -2.1619715330

H 1.9281294051 -0.9231319511 -1.0435906942

H -2.8703231825 -1.8093492044 -0.7197002497

H -2.6473378882 -0.9065204126 0.8114093692

H -2.0236411090 -2.5883562746 0.6510018469

Transition state (TS$_{HT}$)

P -0.0545516629 -0.4012267943 0.0535869290

C -1.8001011589 1.3640994946 -0.2054409019

C -0.4113902226 -2.1220668340 -0.2178741625

C 2.2940845456 0.9754871931 -0.0634646188

O 1.4722002713 -0.2403706636 -0.1770057698

O -0.5452256185 0.1696253971 1.4548485988

O -0.9003663590 0.5561031562 -0.9403936344

H -2.8241209439 0.9900099265 -0.2222275190

H -1.4510676204 1.1270628805 0.9775744119

H -1.6512400144 2.4246373974 -0.3935532229

H -1.4788950261 -2.2959692303 -0.0736528960

H 0.1567492912 -2.7262250402 0.4921919775

H -0.1239109036 -2.3968685336 -1.2347525237

H 3.3174105667 0.6256863782 -0.1486466625

H 2.1217483319 1.4392427595 0.9057334791

H 2.0431759274 1.6428431366 -0.8853864360

Hydrogen transferred (HT) state

P -0.3120900000 -0.1324400000 0.0145200000

C -0.2539700000 2.4432000000 -0.7294800000

C -1.4396500000 -1.4015100000 -0.5098800000

C 2.4318600000 -0.2009800000 0.1027400000

O 1.1129200000 -0.4607400000 -0.5040300000

O -0.1583900000 -0.0184400000 1.5864200000

O -0.9035200000 1.2107500000 -0.5811500000

H -0.9457700000 3.2711400000 -0.7460600000

H -0.8495500000 0.3538200000 2.1525200000

H 0.6780900000 2.4283600000 -1.2791700000

H -2.4587500000 -1.1362400000 -0.2224800000

H -1.1573800000 -2.3472400000 -0.0446700000

H -1.3899500000 -1.5006400000 -1.5958200000

H 3.1272500000 -0.7701600000 -0.5050700000

H 2.4265200000 -0.5516800000 1.1313600000

H 2.6458400000 0.8636400000 0.0468100000

Conical Intersection D_2/D_3, C_s ($\Delta E = 1.89 \cdot 10^{-5}$ a.u.)

P -0.466456 -0.119981 0.002683

C 1.418396 0.158537 1.983725

C -2.137137 0.476377 0.004061

C 1.420642 0.157795 -1.981742

O 0.234896 0.533140 -1.249473

O -0.280867 -1.605354 -0.002017

O 0.231512 0.527467 1.252393

H 1.318620 0.632969 2.944334

H 1.447881 -0.917008 2.070148

H 2.279624 0.540649 1.456536

H -2.640893 0.094153 0.882297

H -2.635811 0.109925 -0.883742

H -2.152042 1.556240 0.014562

H 1.312462 0.613567 -2.950060

H 1.457646 -0.919032 -2.042854

H 2.280838 0.556916 -1.464909

Conical Intersection D_1/D_2, C_s ($\Delta E = 9.373 \cdot 10^{-4}$ a.u.)

P -0.451312 -0.205822 0.000556

C 1.373402 0.166944 2.079336

C -2.101559 0.423571 0.001501

C 1.381738 0.173224 -2.071852

O 0.319677 0.650935 -1.206712

O -0.179447 -1.659467 -0.007450

O 0.334454 0.663466 1.196524

H 1.379723 0.885129 2.882914

H 1.147954 -0.826956 2.412937

H 2.310593 0.204132 1.547215

H -2.615692 0.055907 0.881129

H -2.616050 0.057392 -0.878810

H -2.099485 1.504075 0.002540

H 1.321658 0.844472 -2.912778

H 1.223925 -0.848318 -2.349645

H 2.317142 0.314243 -1.557151

Conical Intersection D_2/D_3, C_1 ($\Delta E = 1.236 \cdot 10^{-3}$ a.u.)

P -0.220993 0.203060 -0.001305

C 0.058367 -1.070679 2.389501

C 0.217739 -0.947775 -2.396893

C 0.524840 1.808496 -0.328543

O 0.620606 -0.231585 1.371299

O -1.683497 0.110818 -0.069833

O 0.593422 -0.861124 -1.015545

H 0.183609 2.512498 0.415893

H 0.192420 2.134382 -1.305640

H 1.601306 1.727801 -0.302332

H 0.723661 -1.836844 -2.732106

H 0.607652 -0.090113 -2.926984

H -0.849871 -1.009697 -2.490973

H 0.776407 -1.097181 3.188974

H -0.908129 -0.694758 2.695925

H -0.062836 -2.057770 1.942901

Conical Intersection D_1/D_2, C_1 ($\Delta E = 1.0914 \cdot 10^{-4}$ a.u.)

P -0.143468 0.333941 0.109633

C 0.015665 -1.182338 2.301583

C 0.160354 -1.057750 -2.315002

C 0.589846 1.900973 -0.377179

O 0.435360 -0.072257 1.458283

O -1.641966 0.322339 -0.093608

O 0.683829 -0.742776 -0.991031

H 0.219640 2.637827 0.325470

H 0.291973 2.181016 -1.376382

H 1.664032 1.824946 -0.298938

H 0.794787 -1.842769 -2.680661

H 0.247140 -0.162939 -2.916686

H -0.869477 -1.354023 -2.212642

H 0.633224 -1.117359 3.174716

H -1.028942 -1.087297 2.542172

H 0.197856 -2.109362 1.772909
